# Modulation of Inflammation by Plant-Derived Nutraceuticals in Tendinitis

**DOI:** 10.3390/nu14102030

**Published:** 2022-05-12

**Authors:** Anna-Lena Mueller, Aranka Brockmueller, Ajaikumar B. Kunnumakkara, Mehdi Shakibaei

**Affiliations:** 1 Musculoskeletal Research Group and Tumor Biology, Chair of Vegetative Anatomy, Faculty of Medicine, Institute of Anatomy, Ludwig-Maximilian-University Munich, Pettenkoferstr. 11, 80336 Munich, Germany; a.mueller@med.uni-muenchen.de (A.-L.M.); aranka.brockmueller@med.uni-muenchen.de (A.B.); 2 Cancer Biology Laboratory and DBT-AIST International Center for Translational and Environmental Research (DAICENTER), Department of Biosciences and Bioengineering, Indian Institute of Technology (IIT) Guwahati, Guwahati 781039, India; kunnumakkara@iitg.ac.in

**Keywords:** tendinitis, tendinopathy, nutraceuticals, polyphenols, tissue engineering, inflammation, tendon

## Abstract

Tendinitis (tendinopathy) is a pro-inflammatory and painful tendon disease commonly linked with mechanical overuse and associated injuries, drug abuse, and lifestyle factors (including poor diet and physical inactivity) that causes significant healthcare expenditures due to its high incidence. Nuclear factor kappa B (NF-κB) is one of the major pro-inflammatory transcription factors, along with other inflammation signaling pathways, triggered by a variety of stimuli, including cytokines, endotoxins, physical and chemical stressors, hypoxia, and other pro-inflammatory factors. Their activation is known to regulate the expression of a multitude of genes involved in inflammation, degradation, and cell death. The pathogenesis of tendinitis is still poorly understood, whereas efficient and sustainable treatment is missing. Targeting drug suppression of the key inflammatory regulators represents an effective strategy for tendinitis therapy, but requires a comprehensive understanding of their principles of action. Conventional monotherapies are often ineffective and associated with severe side effects in patients. Therefore, agents that modulate multiple cellular targets represent therapeutic treatment potential. Plant-derived nutraceuticals have been shown to act as multi-targeting agents against tendinitis via various anti-oxidant and anti-inflammatory mechanisms, whereat they were able to specifically modulate numerous signaling pathways, including NF-κB, p38/MAPK, JNK/STAT3, and PI3K/Akt, thus down-regulating inflammatory processes. This review discusses the utility of herbal nutraceuticals that have demonstrated safety and tolerability as anti-inflammatory agents for the prevention and treatment of tendinitis through the suppression of catabolic signaling pathways. Limitations associated with the use of nutraceuticals are also described.

## 1. Introduction

**Figure 1:**
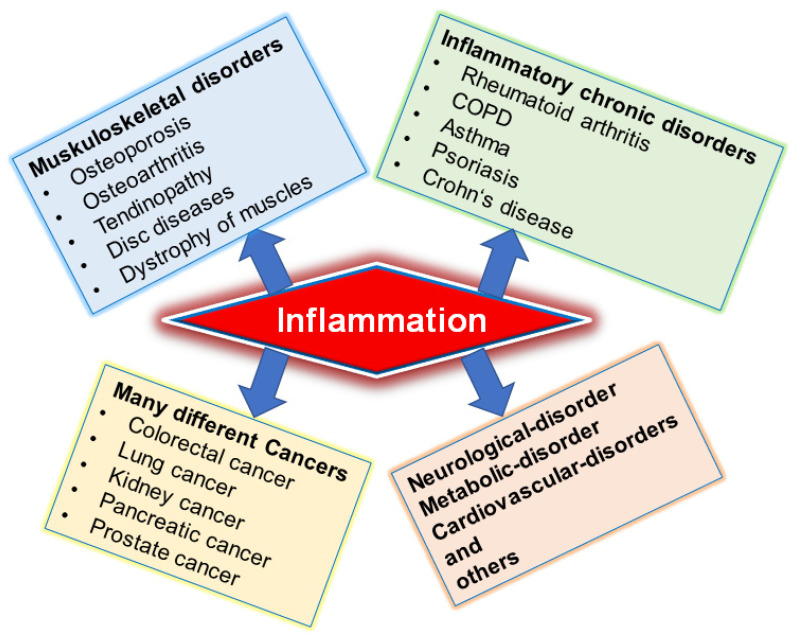
Inflammation in the context of different chronic disorders.

Tendinopathy is a frequently occurring and very complex disease, restricting the daily lives of many people worldwide due to symptoms such as severe pain and immobility [1,2,3,4]. The reasons for development of tendinopathies or tendon injuries are diverse, ranging from tendon rupture and trauma-associated damage to mechanical stress and overuse to lifestyle factors (including poor diet or lack of exercise), accompanied by inflammation and inflammation-linked processes [5,6,7,8]. It is well known that inflammation is a major cause of many chronic diseases, including cancer, neurological, metabolic, cardiovascular, and skeletal disorders, including tendinitis among many others (Figure 1). Therefore, targeting inflammation is fundamental to the prophylaxis, therapy, and treatment of tendinitis [9]. Moreover, one of the main players in cellular inflammatory processes is the master pro-inflammatory transcription factor, nuclear factor kappa B (NF-κB), which is triggered by different cytokines, such as the tumor necrosis factor (TNF)-α, TNF-β, or interleukin (IL)-1β, and, when activated, leads to further expression and up-regulation of pro-inflammatory genes and catabolic enzymes, contributing to the pathogenesis of tendinitis by further fuelling inflammation and tissue degradation in tendon [10,11,12]. So, based on the fact that NF-κB and other signaling pathways, such as p38/MAPK, JNK/STAT3, and PI3K/Akt, are known to regulate more than 500 genes involved in inflammation and associated processes, they demonstrate potential targets to lower the level of inflammation in tendinitis treatment, which is currently still lacking in efficiency or safety [10,12,13,14,15,16,17,18].

Current standard treatment strategies of tendinitis mainly follow the application of oral non-steroidal anti-inflammatory drugs (NSAIDs) and glucocorticoids [19,20], whereas NSAIDs mainly act as analgesics and inflammation-targeting agents, inducing the inhibition of cyclooxygenase (COX) and prostaglandin endoperoxide synthase enzymes as main pharmacological targets that are regulators of prostaglandin biosynthesis, which in turn are strongly involved in inflammation [21,22]. Glucocorticoids also fight inflammation through diverse immune-suppressive and anti-inflammatory actions, such as downregulating signal transduction downstream of several pro-inflammatory pathway receptors, inhibiting lymphocyte activity, and proliferating or modulating the innate immunity [23,24]. Both NSAIDs and glucocorticoids frequently lead to severe side effects in patients [21,22,25], including organ damages of the gastrointestinal tract, as well as hepatic, renal, cardiovascular, or pulmonary complications [22,26]. In addition, tendon’s ability to regenerate, partly via the synthesis of proteoglycans and collagen, as major constituents of tenocytes and their extracellular matrix (ECM), has been found to be greatly reduced following application of NSAIDs and corticosteroids [27,28,29,30]. This clearly underscores the need for clinically safe and efficacious new anti-inflammatory agents.

In contrast, several phytopharmaceuticals have been shown to be nontoxic, clinically and pharmacologically safe, and suitable for screening as potential therapeutic metabolites [31]. In a phase I clinical trial, of patients with high-risk or pre-malignant lesions with the chemopreventive agent curcumin **[32],** it was found to be very potent in anti-inflammatory effects in many chronic diseases by promoting modulation of various signaling pathways and cellular mechanisms associated with inflammation, degradation, and apoptosis [15,33,34,35,36], including different types of cancer [37,38,39,40], neuro-inflammatory disorders [41,42,43], chronic destructive pulmonary disease (COPD) [44,45,46,47], psoriasis [48,49,50], cardio-vascular disease [51,52,53], and many more. In addition, natural products showed to have a restorative effect on skeletal disorders, such as osteoarthritis (OA), osteoporosis, intervertebral disc disease, and rheumatoid arthritis (RA), while representing a safe treatment method [54,55,56,57,58,59,60]. In fact, even tendinitis as a major health concern, due to the lack of effective and safe treatment, has been revealed to be positively affected by the effects of phytochemicals [36,61,62,63,64,65]. Given this background, together with the strong desire to alleviate symptoms caused by tendinitis, herbal natural compounds represent a promising approach for the treatment, co-treatment, and prevention of inflammatory tendinitis disease [3,4,10,36,62].

In this review, we will present different natural occurring agents that have currently been demonstrated to show efficiency in the treatment of tendinitis and tendon regeneration, describing their individual modes of action to better understand their potential for inflammation-related tendinitis therapy.

## 2. Signaling Pathways in Inflammation and Inflammation-Associated Diseases

As of recent, it is widely known that chronic diseases, such as RA, inflammatory bowel diseases (IBD), different types of cancer, OA, COPD, cardiovascular and neurological disorders, and many more, are mostly associated with inflammation that turned into a long-running condition, exposing the host’s immune system to a permanent state of stress [9,66,67]. Inflammation in chronic diseases is part of the immune response to pathogenic conditions in the human organism that is mediated by various molecular mechanisms and can be triggered by a wide range of conditions, including environmental factors, lifestyle, diet, and overall activity [68]. Major signaling pathways that contribute to an inflammatory environment when being dysregulated are p38/MAPK, IL-6/JAK/STAT3, and PI3K/Akt, as well as the NF-κB signaling pathway [67].

### 2.1. p38/MAPK Signaling Pathway

**Figure 2:**
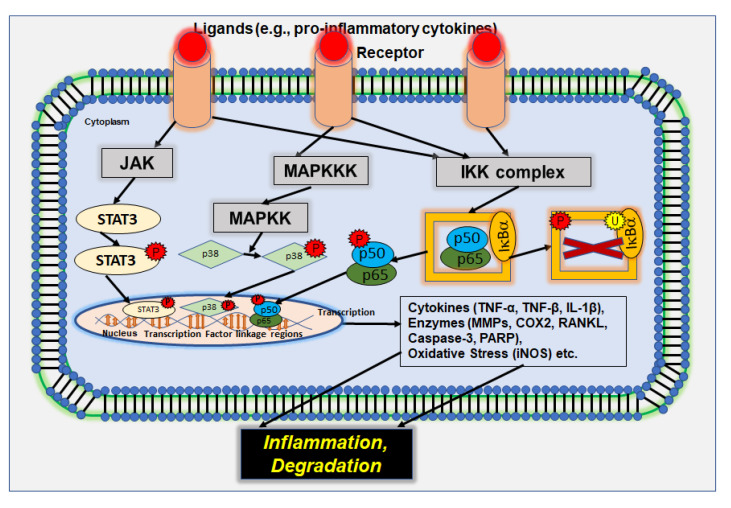
Molecular backgrounds of NF-κB, STAT3, p38/MAPK signaling linking inflammation, and degradation. Under physiological conditions, NF-κB is located as a heterodimeric complex of p50 and p65 bound to its inhibitor IκBα in the cytoplasm. The upstream IKK complex can activate IκBα by its phosphorylation and ubiquitin-dependent degradation, leading to NF-κB release that is then activated (p65- NF-κB) and translocates into the nucleus to activate the transcription of target genes, including cytokines and pro-inflammatory enzymes. The activation of the p38/MAPK signaling pathway by upstream MAPKK is promoted by external stimuli linked to stress and inflammation, binding to various receptors. Activated p38 in turn results in the transcription of several pro-inflammatory genes, such as COX-2, iNOS, MMPs, or RANKL, which are strongly involved in tissue remodeling. Another major signaling pathway involved in inflammation, the JAK/STAT3 pathway, is activated by IL-6, consequently inducing STAT3 phosphorylation and activation, which then translocates to the nucleus, where it regulates the expression of many genes linked to apoptosis, inflammation, and tissue degradation, such as Bcl-X_L_, COX-2, and various cytokines. Abbreviations: COX-2; cyclooxygenase-2, IκBα; NF-κB inhibitor alpha, IKK; IκB kinase, IL-1 β; Interleukin 1 beta, iNOS; inducible nitric oxide synthase, JAK; Janus kinase, MAPKK; MAPK kinase, MAPKKK; MAPKK kinase, MMPs; matrix metalloproteinases, PARP; Poly(ADP-Ribose)-Polymerase, RANKL; receptor activator of NF-κB ligand, STAT3; signal transducer and activator of transcription 3, TNF- α; tumor necrosis factor alpha, TNF- β; tumor necrosis factor beta.

The p38 mitogen-activated protein kinase (MAPK) functions together with other MAPK family members, as a cellular signal transducer for external stimuli, and mediates a wide range of cellular responses. P38/MAPK is stimulated by pro-inflammatory and stressful stimuli, such as TNF-α, IL-1β, or lipopolysaccharides, by binding to and activating various receptors, including G-protein-coupled receptors (GPCRs), toll-like receptors (TLRs), cytokine receptors, growth factor receptors, and other receptors linked to environmental stress [66,67,69]. The upstream activation of p38/MAPK by MAPK kinases (MAPKK) in turn plays a pivotal role in the release of various pro-inflammatory cytokines, such as IL-6, IL-1, IL-8, or TNF-α, and also leads to the induction of inflammation-involved enzymes, such as COX-2 and inducible nitric oxide synthase (iNOS), as well as the activation of matrix metalloproteinases (MMPs) and the modulation of receptor activator of NF-κB ligand (RANKL) expression, which is essential for osteoclastogenesis and bone resorption. Thus, targeting the p38/MAPK pathway demonstrates a promising strategy in the treatment of inflammatory diseases, especially in musculoskeletal disorders, such as OA or osteoporosis, because of RANKL modulating potential (Figure 2) [66,67,70,71,72,73].

### 2.2. IL-6/JAK/STAT3 Signaling Pathway

In another major signaling pathway involved in inflammation, the IL-6/JAK/STAT3 pathway, Janus kinase (JAK), a non-receptor cytoplasmic tyrosine kinase, is activated by the pro-inflammatory cytokine IL-6 that is often found in enhanced levels in chronic disorder patients, finally leading to the phosphorylation and activation of the signal transducer and activator of transcription 3 (STAT3) which, in turn, translocates into the nucleus and regulates expression of genes that are involved in cell differentiation and proliferation among other cellular functions, such as cyclin D, Bcl-X_L_, COX-2, vascular endothelial growth factor (VEGF), and cytokines [67]. The hyperactivation of STAT3 signaling has been observed in most human cancer cases, and abnormal levels of IL-6, stimulating STAT3, are commonly found in patients suffering from chronic disease, such as IBD or RA. Moreover, STAT3 contributes to the promotion of other inflammatory pathways and is highly interconnected with NF-κB signaling, co-regulating a wide range of pro-inflammatory genes (Figure 2) [74,75,76,77].

### 2.3. PI3K/Akt Signaling Pathway

The dysregulation of phosphoinositide-3-kinase (PI3K) signaling represents another key contributor to inflammatory conditions [67]. PI3K transduces upstream signals from receptor tyrosine kinases, GPCRs, cytokine receptors, growth factor receptors, and others by synthesizing the phospholipid phosphatidylinositol 3,4,5-trisphosphate (PIP3), a second messenger protein used by various cell receptors to mediate many cellular processes, including growth, mobility, and differentiation. PIP3 activates a variety of effector molecules, including the serine/threonine protein kinase B (Akt). Akt as a downstream molecule of PI3K promotes further downstream effectors, such as the mammalian target of rapamycin (mTOR), glycogen synthase kinase 3 beta (GSK3β), forkhead box protein O1 (FOXO1), and others, which can regulate several catabolic and anabolic processes to control metabolism, cell growth, and apoptosis [67,78,79,80,81]. Moreover, NF-κB signaling, as a major contributor to inflammation-associated conditions, is activated by PI3K-induced Akt, which is essential for the expression of pro-inflammatory genes, such as TNF-α and IL-1 [14,67].

### 2.4. NF-κB Signaling Pathway

In this context, NF-κB has been found as a major initiator of pro-inflammatory cascades and mechanisms (Figure 2) [82]. Under resting conditions, NF-κB is located as a heterodimeric complex consisting of p50 and p65 in a latent state in the cytoplasm, bound to its natural inhibitor nuclear factor of kappa light polypeptide gene enhancer in B-cells inhibitor alpha (IκBα) [83,84]. As a regulator of the NF-κB signaling pathway, the IκB kinase (IKK) complex can activate IκBα by its phosphorylation and ubiquitin-dependent degradation, releasing NF-κB that is then able to translocate into the nucleus in its active form (p-NF-κB), where it binds to specific consensus sequences and activates the transcription of various target genes (canonical pathway) (Figure 2). Most of the proteins that are encoded by NF-κB target genes are part of the host’s immune response, such as cytokines (e.g., TNF-α, TNF-β, IL1-β), pro-inflammatory enzymes, as well as stress responsive genes (e.g., MMPs, COX-2, RANKL, caspase-3, PARP) [85,86,87,88]. A variety of those genes activated by NF-κB, such as TNF-α, TNF-β, IL-1, and IL-8, in turn activate NF-κB signaling again, building a positive feedback loop by continuously stimulating inflammation and enhancing stress responses [82,87]. Moreover, NF-κB signaling can be activated by many other stimuli, including different growth factors, viral and bacterial antigens, radiation, or reactive oxygen species (ROS), binding to and being recognized by the TNF receptor superfamily or TLRs [82,87,88]. However, NF-κB can also be induced via the non-canonical pathway which is based on processing p100 NF-κB members via the NF-κB-inducing kinase as a central operator, finally generating heterodimers p52 and RelB which translocate into the cell nucleus to induce target gene transcription. The non-canonical NF-κB pathway, which shows a rather slow character compared to the rapid canonical pathway, is mainly induced by TNF ligands binding to their receptor. Most of them also stimulate the canonical pathway of NF-κB initiation and regulate processes that include the functional linkage of both NF-κB pathways. However, dysregulation of the non-canonical NF-κB pathway has also been demonstrated to play a crucial role in the pathogenesis of inflammatory diseases [12,86,89]. Therefore, one main target to fight inflammation is modulating the NF-κB signaling pathway, which is known as the master regulator of inflammation and holds sway over many molecular processes [12,83,88,90].

## 3. Plant-Derived Nutraceuticals and Inflammation

In recent decades, nutraceuticals, i.e., so-called functional foods, have gained increasing interest worldwide. This was not only due to their great health-promoting anti-inflammatory potential, but also due to the low or even no toxic effects in administration compared to standard synthetic medicals [91]. Especially with regard to inflammation and inflammation-associated diseases, such as cancer, RA, IBD, the metabolic syndrome, and cardiovascular or neurological disorders, among many others, various nutraceuticals have been found to reveal inflammation-protective properties [92,93,94,95,96,97,98]. These properties were found in earlier research to rely, at least in part, on the ability of a wide range of polyphenols to target and modulate a number of pro-inflammatory transcription factors, including NF-κB. As NF-κB signaling has been described as a major contributor to inflammation in detail before, the great potential of plant-derived NF-κB targeting nutraceuticals becomes obvious [10,36,54,99,100,101,102,103,104].

As an example, it has been demonstrated that phenolic acids of blueberries exhibited anti-inflammatory effects by targeting NF-κB and suppressing TNF-α and IL-6 production, thus demonstrating great potential in the fight against chronic diseases, such as diabetes, cardiovascular and neurodegenerative disease, and several more [105,106,107]. In addition, targeting NF-κB and STAT3 signaling, as well as pathway-dependent gene end products, is known to be promoted by turmeric extract curcumin, what has been proven in a wide range of different studies investigating on Alzheimer’s disease, cancer, musculoskeletal disorders, or ulcerative colitis, among many others [54,108,109,110,111,112,113,114]. Furthermore, Lactucin, a bio-active component found in endive, chicory, and romaine lettuce, has been shown to target pro-inflammatory STAT3 in order to suppress adipogenesis in obesity and obesity-linked complications [115], whereas NF-κB was blocked by Lactucin in cancer cells [116]. Moreover, other signaling pathways that have been described to play an essential role in inflammation, such as MAPK, were shown to be modulated by polyphenols, e.g., resveratrol or epigallocatechin-3-gallate (EGCG), which abolished inflammation and ROS by the functional blockade of p38/MAPK in RA [93,117]. In addition, the PI3K signaling pathway has been shown to be inhibited by the flavonoid quercetin with a simultaneous decrease in IL-1β and IL-6 levels, as well as by resveratrol, showing the neuro- and tendinitis-protective effects following down-regulation of the PI3K signaling pathway [14,94,118].

Besides these examples, the range of polyphenols that have been shown to modulate major inflammatory pathways, pathway-dependent effector molecules, and gene expression is huge and include calebin A, kaempferol, anthocyanins, genistein, various phenolic acids, caffeine, allicin, cinnamon polyphenols, and many more [14,36,119,120,121,122,123]. This demonstrates their great potential for preventing and treating the inflammation and pathogenesis of chronic diseases, and explains their great anti-inflammatory properties and anabolic effects [56,124].

In the following section, we will describe the anti-inflammatory activities of polyphenols focusing on tendinitis.

## 4. Plant-Derived Nutraceuticals in the Treatment of Tendinitis

With regard to chronic musculoskeletal disorders, such as RA, osteoporosis, or OA, various polyphenols have been found to reduce inflammation and, in addition, exhibit tissue-protective and regeneration-stimulating effects [36,92,93,125] due to their ability to modulate dysregulated pro-inflammatory pathways back to physiological conditions, and, more so, to stimulate anabolic processes in the respective tissues, helping them to regenerate and heal [36,126,127,128,129]. Additionally, in tendinitis, as a very common and painful condition disabling musculoskeletal functionality and still lacking sustainable treatment, various plant-derived compounds have been demonstrated to affect inflammation and promote tissue healing, thus representing promising bio-active treatment agents in tendinitis therapy [10,14,36,62,65,130,131,132,133]. Since plant-derived nutraceuticals are ingredients of a large number of vegetables, spices, and fruit, they can be easily integrated into people’s daily diet while being cost-effective [134,135,136]. Moreover, many nutraceuticals are available in the form of dietary supplements in various forms, such as pills or sachets. However, poor bioavailability of some compounds is frequently being mentioned, e.g., due to poor absorption ability, fast systemic metabolism, and elimination or low solubility, among other factors that could contribute to the limiting effects of nutraceuticals in their administration [135,137,138]. Nevertheless, due to their high potential in many inflammation-associated diseases, such as tendinitis, it is reasonable to study their pharmacological effects.

### 4.1. Modulation of Inflammatory Pathways in Tendinitis

**Figure 3:**
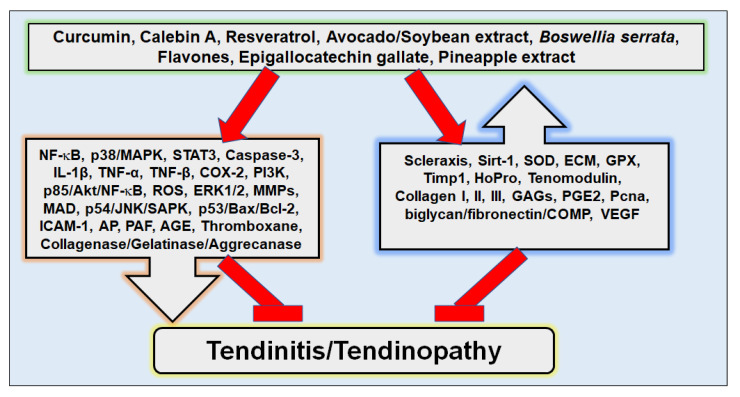
Distinct plant-based nutraceuticals and their regulatory mechanisms for fighting tendinitis. Various polyphenols have been demonstrated to modulate inflammatory mechanisms by downregulating major pro-inflammatory players (e.g., NF-κB, caspase-3, MMPs, PI3K) and upregulating expression of genes required for tendon vitality and proliferation (e.g., Scleraxis, tenomodulin, collagen, Sirt-1), and thereby help to fight tendinitis. Abbreviations: AGE; advanced glycation end-products, Akt; serine/threonine kinase B, AP; alkaline phosphatase, Bax; Bcl-2-associated X protein, Bcl-2; B-cell lymphoma 2, COMP; cartilage oligomeric matrix protein, COX-2; cyclooxygenase-2, ECM; extracellular matrix, ERK; extracellular signal-regulated kinase, GAGs; glycosaminoglycans, GPX; glutathione peroxidase, HoPro; hydroxyproline, ICAM-1; Intercellular Adhesion Molecule 1, IL-1 β; Interleukin 1 beta, JNK; c-Jun N-terminal kinase, MAD; mitotic arrest deficient, MAPK; mitogen-activated protein kinase, MMPs; matrix metalloproteinases, NF-κB; nuclear factor kappa B, PAF; platelet-activating factor, Pcna; proliferating cell nuclear antigen, PGE2; prostaglandin E2, PI3K; phosphoinositide-3-kinase, ROS; reactive oxygen species, SAPK; stress-activated phospho-kinases, Sirt-1; Sirtuin-1, SOD; superoxide dismutase, STAT3; signal transducer and activator of transcription 3, Timp1;tissue inhibitor of metalloproteinase 1, TNF- α; tumor necrosis factor alpha, TNF- β; tumor necrosis factor beta, VEGF; vascular endothelial growth factor.

In tendinitis, as in other chronic diseases, inflammatory pathways and mediators are abnormally activated, which leads to further fuelling of the inflammatory environment, and finally to the chronification and degeneration of tissue [4,139,140]. Therefore, targeting these biological pathways underlying inflammation is assumed to be a very effective strategy to break through catabolic events in tendon tissue [10,56,141,142]. As of recent, various polyphenols have been proven to show inflammation-protective activity in disordered tendon tissue, including compounds of turmeric, calebin A and curcumin, green tea extracts, various flavones, pineapple extract bromelain, boswellia acid, and resveratrol, among several others [10,14,36,130,143,144,145]. The main target pathways that have been found to be modulated by nutraceuticals specifically in tendinitis are NF-κB, STAT3, PI3K, and associated pathway dependent gene products (such as MMPs, COX-2, and caspase-3), as well as stimulated cytokines, including IL-1β, TNF-α, and TNF-β (Figure 3) [10,36,132,146,147]. With nutraceuticals targeting NF-κB, translocation from cell cytoplasm into the nucleus is suppressed, inhibiting its activation. As a consequence of NF-κB being inactivated, further downstream cascades, leading to inflammation and apoptosis, are down-regulated as well. For example, in tendinitis, Bcl-2 and Bax have been found to be affected, which play an essential role in cell apoptosis [10]. In addition, cytokines stimulating NF-κB, and, in turn, being promoted by NF-κB such as TNF-α, TNF-β, or IL-1β, were shown to be inhibited, whereas the activity of other apoptosis-promoting enzymes, such as caspase-3, was also found to be down-regulated by nutraceuticals in tendinitis, probably due to NF-κB modulation [14,54,148,149]. In the context of nutraceutical-induced NF-κB modulation, COX-2 inhibition as well as alterations in MMP activity patterns have also been observed in tendon, which are not only of high relevance for inflammation, but also for tissue remodeling processes, making them another great target in tendon regeneration therapy [146,149,150,151,152].

In addition, it has been observed that with the down-regulation of inflammatory pathways, simultaneously anabolic cascades, such as tendon-specific transcription factor scleraxis promotion, can be stimulated by nutraceuticals that act in tandem, thus demonstrating the great healing potential for tendinitis [36]. Overall, the number of cascades originated by the major players of inflammation, such as NF-κB, STAT3, PI3K, and MAPK, shows the great potential in targeting them in inflammation-associated diseases, such as tendinitis, which certain nutraceuticals have already been shown to be capable of.

### 4.2. Plant-Derived Compounds Proposed for Tendinitis Treatment

**Figure 4:**
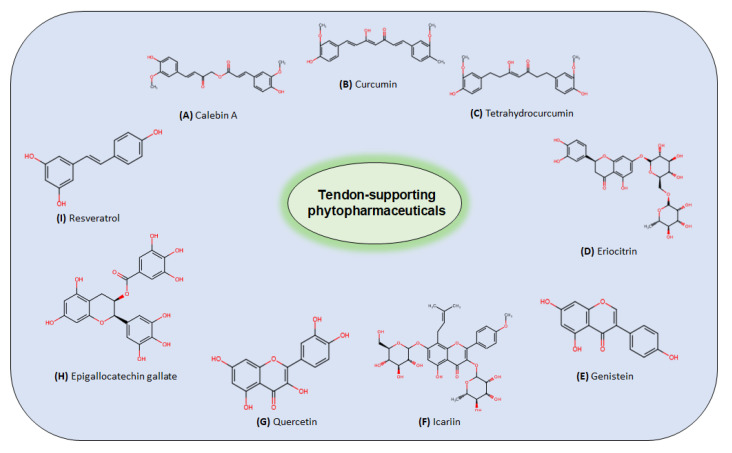
Chemical structures of tendon-supporting phytopharmaceuticals: curcuminoids (**A**–**C**), flavones/flavonoids (**D**–**G**), green tea extracts (**H**), and resveratrol (**I**).

Within this chapter, we will describe different natural compounds and polyphenols that have been studied in the context of tendinitis, tendon tissue, and inflammation (Table 1). The chemical structures of some compounds are shown in Figure 4.

#### 4.2.1. Avocado/Soybean Unsaponifiables

Avocado and soybean unsaponifiables (ASUs) are made of oils extracted from fruit and seeds of avocado and soybean that exhibit active anti-inflammatory properties; thus, they are recommended as supplementary treatment in inflammation-linked chronic diseases, such as OA [153,154,155,156]. In a recent study by Grzanna et al., the effects of ASU have been examined in IL-1β-induced inflammation horse tenocytes. ASUs were concomitantly administered with glucosamine (GLU) and chondroitin sulfate (CS), because this mixture has been used in osteoarthritis joint-inflammation therapy before. As a result of the study, it has been demonstrated that ASU was able to significantly suppress IL-1β-induced inflammation and to reduce COX-2 and prostaglandin E2 expression, indicating that ASU can act as a potential treatment agent in tendon-associated inflammation for combination therapy with GLU and CS (Table 1) [157]. However, because of the small number of studies conducted with ASU in the context of tendinitis, these results must be considered as an only vague hint of ASU’s potential in tendinitis treatment and further research has to be carried out.

#### 4.2.2. Bromelain

Bromelain is a natural complex of proteolytic enzymes that are derived from fruit or stem of the *Ananas cosmosus* (pineapple). It has been used as another phytopharmaceutical in folk medicine for centuries due to its anti-inflammatory, anti-cancer, immune-modulating, as well as anti-thrombotic properties, to name just a few, as well as its safety for administration [158,159,160]. In musculoskeletal injuries or disorders, it is also known to reduce acute pain and swelling [161]. In a study by Aiyegbusi et al., the effects of aqueous extracts of different parts of the pineapple plant were investigated on tenocyte proliferation in rats in vivo after crush injury of the Achilles tendon. In addition, the tendon malondialdehyde (MDA) level, which is a marker of oxidative stress, was analyzed [161,162]. Finally, the extracts of pineapple flesh and bark were found to promote the proliferation of tenoblasts, comparable to untreated tendon, whereas leaves and core extracts negatively affected the proliferation of tenocytes. Using the pineapple flesh extract, even the MDA level in tendon could be alleviated, suggesting that the anti-oxidant properties of the pineapple are located in its flesh, while both flesh and bark showed the potential to promote tendon regeneration and injury healing by promoting tenoblast proliferation [161]. The proliferation-stimulating properties of bromelain in tendon were supported by further studies in rats, arguably due to the role for ROS, or, on the other hand, the increased expression of platelet activating factor (PAF) by modulating the cytokine system, leading to the proliferation of tenoblasts [130,163].

#### 4.2.3. Curcuminoids

Curcuminoids (Figure 4) describe a group of bio-active nutraceutical compounds found in the rhizome of turmeric (*Curcuma longa*) that exhibit considerable inflammation-protective and anti-oxidant properties and have a long history in traditional Ayurvedic and Chinese medicine [34,35,164,165,166].

##### Curcumin

Curcumin is the most abundant polyphenolic compound found in the group of curcuminoids that gives turmeric its characteristic intensive yellow colour [167]. Due to its multiple health-beneficial effects in a wide range of chronic diseases, including RA, OA, cancer, neurological, cardiovascular or respiratory disorders, it has reached a lot of attention as an anti-inflammatory agent in therapy [35,54,111,127,167]. As a well-known curcuminoid, curcumin has also been widely investigated in the context of tendon and tendinitis in several studies, where it was proven to stimulate tendon vitality and regeneration [10,64,131,146,168,169,170,171]. For instance, in previous in vitro research using human tenocyte cultures, it was found that curcumin was able to suppress NF-κB activation triggered by IL-1β through inhibition of IκBα and NF-κB -dependent pro-inflammatory COX-2 and MMPs as well as Bcl-2, Bcl-xL and TRFA-1 that are involved in apoptosis. Moreover, it was demonstrated that these effects were at least partly executed by down-regulation of PI3K/Akt signaling promoted by curcumin, suggesting the polyphenol as an effective treatment option for tendinitis as well as for prophylaxis by its modulation of pro-inflammatory NF-κB signaling [10]. The strong anti-inflammatory effect of curcumin was also found in a recent in vivo study by Chen et al., demonstrating the down-regulation of pro-inflammatory ROS, TNF-α, IL-1β and MMPs in rats during curcumin treatment in the form of curcumin/Mg^2+^ hydrogels as well [146].

In addition, in a study by Sajithlal et al. investigating the preventive and therapeutic potential of curcumin in tail tendon of diabetic rats, it has been demonstrated that curcumin treatment significantly reduced oxidative stress by the suppression of lipid peroxidation and even more, prevented increased accumulation of advanced glycation end products and collagen crosslinking in tendon as an issue coming along with diabetes [168]. In another study, of diabetic rats similar observations were made using tetrahydro-curcumin (a metabolite of curcumin) for treatment [172], showing curcumins potential in tendon-associated therapy as an effective anti-oxidant [168,172]. Besides its anti-inflammatory effects, curcumin has also been proven in vitro (human tenocytes) and in vivo (rats) to help in tendon regeneration and healing by promoting collagen I and II synthesis [10,170] and their organization as filaments enhancing tendons biomechanical traits [64] as well as by suppressing the peritendinous adhesion of inflammatory products [169]. Moreover, curcumin has been shown to prevent tendon calcification in rats as a common issue of late-stage tendinitis due to aberrant tendon stem and progenitor cells osteogenic differentiation triggered by inflammation, by promoting tenogenesis while suppressing osteogenesis at the respective pathological sites [131], as summarized in Table 1.

##### Calebin A

Besides curcumin, another bio-active compound of turmeric, namely calebin A, has been recently investigated in in vitro tendinitis study models by our group. Within our study, calebin A was demonstrated with the ability to suppress inflammatory conditions in tenocytes by inhibiting the NF-κB signaling pathway and its associated gene end products, such as COX-2, MMP-9, and caspase-3, which are responsible for matrix degrading and apoptotic processes, leading to tissue inflammation and degeneration. By suppressing these catabolic events, calebin A helps in inflammation protection and tendon regeneration, which was shown by an increased expression of tendon-specific transcription factor scleraxis, tenomodulin, as well as collagen I. The down-regulation of NF-κB with the simultaneous up-regulation of tenogenic scleraxis indicates a multi-modulatory effect of calebin A in tendinitis by targeting the NF-κB–scleraxis axis (Table 1) [36].

Therefore, calebin A represents another powerful nutraceutical with great potential in tendinitis therapy, as well as in its prevention. However, further studies are needed to examine the full potential of the natural compound calebin A in fighting inflammatory and degrading processes in tendinitis [36].

#### 4.2.4. Green Tea Extracts (Epigallocatechin Gallate)

Epigallocatechin gallate (Figure 4), as the most abundant polyphenol component of green tea, has been shown to be beneficial in many different diseases, including cancer, the metabolic syndrome, and neurodegenerative disorders, because of its anti-oxidant and anti-inflammatory characteristics [173,174,175]. These effects were also found in a study investigating human tendon-derived fibroblasts in vitro, in which EGCG administration suppressed IL-1β-stimulated collagenase and stromelysin, as well as the expression of MMPs, and simultaneously reduced the stimulation of p54/JNK/SAPK phosphorylation. Altogether, these outcomes suggest ECM breakdown as an important target for EGCG and other green tea polyphenols, thus proposing a potential target for EGCG therapy in tendon injury [148]. In addition to that, green tea extract has also been shown to remarkably reduce glycation, i.e., the resulting formation of advanced glycation end products (AGE), as well as crosslink collagen in the tail tendon of diabetic rats. These outcomes demonstrate the therapeutic potential of green tea extracts in treating diabetes-related tendon glycation, since the formation of glycation and AGE in tendon finally leads to impaired tendon turnover, making it even more vulnerable to tendinitis [145]. Furthermore, in an in vivo study by Rutter et al., it has been demonstrated that green tea polyphenols could prevent collagen aging markers from rising and to even delay collagen crosslinking by anti-oxidant mechanisms in rats, further supporting green tea extract as a promising anti-glycation agent. These effects could be even enhanced by the concomitant supplementation of vitamin C and E [176]. Besides their anti-oxidant mechanisms, green tea polyphenols are able to stimulate recovery processes during tendinitis, as shown by the promotion of ECM components and glycosaminoglycans, among other recovery elements after Achilles tendinitis in rats, in combination with a glycine diet. In addition, the synthesis of collagen I as a crucial constituent of tendon was remarkably enhanced by green tea treatment. Simultaneously, inflammatory pathways were modulated in the form of decreased MMP-9 levels, which is important for the remodeling of tissue, whereas MMP-2 and IL-1β, both involved in the active remodeling process, remained elevated during green tea treatment, indicating the potential of green tea polyphenols to accelerate tendon tissue remodeling and the regeneration after tendinitis [132,177]. Altogether, several studies indicate that green tea extracts and EGCG represent promising agents when it comes to fighting ROS and inflammation-triggering stimuli, as well as inflammation itself in tendon tissue (Table 1). However, further research and clinical trials have to be conducted in the future for validation purposes.

#### 4.2.5. Flavonoids/Flavones

Flavonoids and flavones (Figure 4) comprise a group of secondary metabolites that are present in a variety of plants, such as celery, parsley, red pepper, chamomile, mint or *gingko bilboa*, and citrus fruit, and are known to exhibit anti-inflammatory and associated properties in a wide range of chronic diseases [178,179,180,181].

##### Anthocyanin

In an in vitro study model of rotator cuff-derived tenofibroblasts, the effect of flavonoid anthocyanin extracted from the black soybean (*Glycine max* (L.) MEER) as anti-oxidants was observed, thus reducing apoptosis in oxidation-stressed tenofibroblasts. Moreover, the suppression of activation of ERK1/2 and JNK accompanied by decreased ROS levels has been assumed to be an important mechanism in the effect of anthocyanins, as, in contrast, ERK1/2 and JAK were up-regulated in H_2_O_2_-induced apoptotic tenofibroblast cultures without anthocyanin treatment, indicating anthocyanins therapeutic potential in rotator cuff tendon that is exposed to oxidative stressors (Table 1) [133].

##### Eriocitrin

In previous research by Shang et al., eriocitrin, as another flavonoid derived from lemon and limes, was investigated for its potential as a bio-active reagent in tendon stem cells in vitro. Eriocitrin that has been described as the most potent anti-oxidant in citrus fruits was found to stimulate tendon stem cell proliferation and to enhance their migration activity, as an important feature of tissue healing and regeneration [147,182,183]. In addition, not only could pro-apoptotic caspase-3 activity be reduced in a concentration-dependent manner, but also the extent of scar formation, determined by scar-formation-related markers fibronectin and biglycan, could be markedly reduced in tenocyte stem cells when treated with eriocitrin, making it another promising plant-derived agent in tendinitis therapy (Table 1) [147].

##### Genistein

In the context of estrogen deficiency, as experienced in menopause, which consequently promotes a loss of collagen and increases the risk of tendinopathies, the flavone and phyto-oestrogen genistein was investigated on collagen synthesis and Achilles tendon in ovariectomized rats in previous research [184,185,186]. Ramos et al. found evidence that genistein was able to prevent collagen loss in the Achilles tendon of rats in a postmenopausal state, underlining its potential for reducing tendinitis risk in women undergoing estrogen deficiency [184]. Moreover, it has been recently demonstrated that genistein improved functional features of Achilles tendon in estrogen-deficient rats, mostly by modulating proliferation-related gene expression (tenomodulin, proliferating cell nuclear antigen (Pcna)), as opposed to collagen remodeling (Table 1) [185]. However, further studies have to be conducted in the future to verify this hypothesis.

##### Icariin

Icariin is the most abundant flavonoid in horny goat weed (*Epimedium grandiflorum*) and has been demonstrated to positively affect bone metabolism, regeneration, and density [187,188]. In a study by Ye et al., flavonoids have been shown to also support the healing and repair of tendon after rotator cuff reconstruction in rats in vivo, mainly by stimulating the synthesis of collagen type I and II. Moreover, the inhibition of bone loss and the promotion of osteogenesis and angiogenesis, as shown by vascular staining, clearly demonstrating enhanced CD31 (platelet endothelial cell adhesion molecule) and VEGF expression as a sign for intrinsic neovascularization around the tendon insertion site through icariin treatment, are assumed to be crucial components for tendon-bone healing (Table 1) [189]. On this background, icariin represents another flavonoid with therapeutic potential when it comes to tendinitis and tendon-associated issues, but still, cellular and molecular mechanisms have to be investigated in the future.

##### Quercetin

The polyphenolic flavonoid quercetin that is found in fruit and vegetables shows unique bio-active properties, such as anti-cancer, anti-inflammation, anti-oxidant, and anti-viral effects, which thus might play a role in health promotion and disease prevention, as well as in treatment [190,191,192]. Interestingly, previous studies have demonstrated that anti-oxidation methods reducing ROS could reduce the extent of tendon adhesion [193,194]. Furthermore, in a recent in vivo study by Liang et al., it has been demonstrated that quercetin treatment of rat tendon adhesion models can lead to increased anti-oxidant enzyme activity, as shown by enhanced levels of glutathione peroxidase and superoxide dismutase, while MDA levels were reduced in a concentration-dependent manner. In addition, histological analysis showed a lower extent of tendon adhesion in the rats treated with higher concentrations of quercetin, and no side effects or toxicity of quercetin therapy were observed [195].

Furthermore, Semis et al. found proof of quercetin exhibiting anti-inflammatory, anti-apoptotic, and anti-oxidant activity on rat tendon in vivo after collagenase-induced tendinitis. Moreover, inflammatory markers such as MMPs, ICAM-1, and STAT3 were activated by tendinitis induction, whereas all of them could be remarkably suppressed by quercetin administration, making it a promising agent for tendon damage protection [149]. In a study by Fu et al., quercetin has been investigated in combination with kaempferol and isorhamnetin as total flavones of sea buckthorn (*Hippophae rhamnoides*) for the healing of patellar tendon in rats. Within the study, it has been observed that the administration of the flavones not only improved the stress of healing tendons, especially at early stages, but also fiber alignment, collagen deposition, healing, and the recovery of the patellar tendon, suggesting that they could be effective for the improved recovery of tendon injuries (Table 1) [144].

#### 4.2.6. Resveratrol

Resveratrol (Figure 4) is a polyphenolic compound that is found in a variety of plants, including grapes, peanuts, or mulberries. It has been reported as a health-beneficial agent with pharmacological activity positively affecting inflammation, ROS, cancer, diabetes, obesity, and other chronic diseases [117,128,196,197,198,199,200,201]. Moreover, resveratrol has been found to play a role in the modulation of a wide range of cellular mechanisms and pathways, including growth inhibition, proliferation and differentiation, apoptosis, and inflammation cascades [14,62,71,202]. In addition, resveratrol, although its mode of action is not completely understood yet, is known to be a potent activator of Sirt-1; thus, it has a modulatory impact on the Sirt-1 signaling pathway [71,203,204] and acts as NF-κB inhibitor [101,205,206]. Furthermore, in the context of tendinitis, the potential of resveratrol to inhibit the NF-κB signaling pathway, induced by IL-1β and pro-inflammatory gene end products linked with it, has been observed in human tenocytes in vitro. Similarly, IL-1β-promoted PI3K activation has also been described to be suppressed in a dose-dependent manner by resveratrol treatment in tenocytes, comparable to the effects of PI3K inhibitors, suggesting PI3K as a main target signaling pathway of resveratrol in order to suppress NF-κB [14]. These anti-inflammatory effects of resveratrol in tenocytes are assumed to be partly associated with the linkage of Sirt-1 and scleraxis, as well as the deacetylation of NF-κB and PI3K [14,62]. In the same context, it has been demonstrated that resveratrol not only exhibited anti-inflammatory properties, but also stimulated the synthesis of collagens, tenomodulin, and tendon-specific transcription factor scleraxis, which is necessary for tissue vitality and regeneration [14]. The healing capabilities of resveratrol in tendon tissue have also been revealed in diabetic rats in vivo suffering from poor wound healing, following enhanced collagen production, vascular proliferation, and higher fibroblast density, which supported the healing process of Achilles tendinitis despite diabetic conditions [61]. Interestingly, in another in vitro study by Busch et al., the previously mentioned target of resveratrol, Sirt-1, was down-regulated in human tenocytes, leading to the expression of apoptotic proteins (Bax, caspase-3), the acetylation of p53 tumor suppressor Akt activation, and scleraxis suppression. Resveratrol could only inhibit IL-1β-induced NF-κB activation to down-regulate inflammatory mediators, such as COX-2 and MMP-9 in tenocytes, when Sirt-1 was expressed [62]. The role of Sirt-1 in association with resveratrol’s anti-inflammatory effects has also been demonstrated in an in vivo study by Poulsen et al. whereby resveratrol prevented dexamethasone-induced senescence of tenocytes triggered by Sirt-1 inhibition [207]. As glucocorticoids are known to promote tendon senescence and collagen attenuation, the effect of resveratrol to activate Sirt-1, thus inhibiting inflammatory and tendon degrading processes, despite glucocorticoid treatment, is of great potential [29,30,208].

Moreover, in a recently conducted study, polydatin, a derivate of resveratrol, was found to exhibit anti-glycation effects in rat tail tendon in vitro. Glycation leads to AGE formation that causes molecular cross-linking of collagen, making it develop resistance to MMPs, leading to strongly reduced collagen turnover, in turn making collagen and tendon less flexible, and increasing the risk of tendinitis. AGE formation has been found to be accelerated by ROS; hence, resveratrol as a plant-derived anti-oxidant could be a potential natural anti-glycation agent for diabetes and could help to prevent diabetes accompanying disorders, such as tendinitis (Table 1) [209].

**Table 1:** Tendon-supporting effects of phytopharmaceuticals.

Agent	Origin	Type of Trial	Presumed Modulation	Mode of Action	Concentration/Dose Range	Reference
**Avocado/Soybean unsaponifiables (ASU)**	**avocado and soybean oils**	in vitro, horse tenocytes	IL-1β COX-2 PGE2	Avocado/soybean unsaponifiables significantly inhibited inflammation response, such as combination therapy with glucosamine and chondroitin sulfate.	8.3 μg/mL of ASU	[157]
**Boswellia acid**	***Boswellia serrata***	in vivo, Achilles tendinitis patients	-	Boswellia acid (as Casperome^®^) showed pain reduction on a visual analogical scale when Casperome^®^ was administered in addition to physical therapy in patients with Achilles tendinitis.	250 mg of Casperome^®^ for 15 and 30 days	[210]
		in vivo, joint inflammation patients	-	Boswellia acid (as Casperome^®^) supplementation accompanied by standard therapy reduced pain and inflammation in knee joints and tendon of rugby players.	500 mg of Casperome^®^ for 5 days, then 250 mg for 23 days	[211]
		in vivo, supraspinatus injury patients	-	*Boswellia serrata* and *Curcuma longa* extracts (as Tendisulfur^®^) reduced pain after arthroscopic supraspinatus tendon repair compared to placebo treatment.	2 daily sachets Tendisulfur^®^ for 15 days, then 1 daily sachet for 45 days	[212]
		in vivo, tendinopathy patients	-	*Boswellia serrata* and *Curcuma longa* extracts alleviated the symptoms (pain and functional limitation) of patients with tendon disease when applied as combinational therapy.	2 tablets twice a day for 1 month	[65]
		in vivo, rotator cuff tendinopathy, Achilles tendinopathy, and lateral epicondylitis patients	-	*Boswellia serrata*, *Curcuma longa* and bromelain extracts (with methyl-sulfonyl-methane, hydrolysed collagen I and II, L-arginine, L-lysin, vitamin C, chondroitin sulfate, glucosamine, and myrrh as Tendisulfur^®^ Forte), in combination with extracorporeal shock wave therapy, accelerated pain relief and remarkably reduced NSAID intake of patients.	2 daily tablets of Tendisulfur^®^ Forte for 1 month, then once a day for a month	[213]
**Bromelain**	**pineapple extracts,** ***Ananas cosmosus***	in vivo, Sprague–Dawley rats	MDA ROS	Pineapple flesh extract stimulated tenoblast proliferation and thus tendon healing after Achilles tendon injury.	30 mg/kg of pineapple flesh axtract for 14 days	[161]
		in vivo, Sprague–Dawley rats	ROS PAF	Pineapple extract bromelain shifted the thromboxane–prostacyclin ratio towards prostacyclin and increased the tenocyte population after Achilles tendon injury.	7 mg/kg of bromelain for 14 days	[130,163]
		in vivo, Achilles tendinopathy patients	-	Pineapple extract bromelain (as dietary supplement Tenosan with arginine, collagen, vitamin C, methyl-sulfonyl-methane, Vinitrox^TM^) boosted the efficacy of extracorporeal shock wave therapy, resulting in better functional and clinical outcome, compared to placebo treatment.	2 daily drug sachets containing 50 mg of bromelain for 60 days	[214]
		in vivo, rotator cuff tendinopathy patients	-	Pineapple extract bromelain (as dietary supplement Tenosan with arginine L-alpha-ketoglutarate, methyl-sulfonyl-methane and hydrolysed collagen I) reduced pain and improved repair integrity of rotator cuff repair.	2 daily drug sachets containing 50 mg of bromelain for 3 months	[215]
		in vivo, rotator cuff tendinopathy, Achilles tendinopathy, and lateral epicondylitis patients	-	Pineapple extract bromelain (with methyl-sulfonyl-methane, hydrolysed collagen I and II, L-arginine, L-lysin, vitamin C, chondroitin sulfate, glucosamine, *Curcuma longa, Boswellia serrata*, and myrrh as Tendisulfur^®^ Forte), in combination with extracorporeal shock wave therapy, accelerated pain relief and remarkably reduced NSAID intake of patients.	2 daily tablets of Tendisulfur^®^ Forte for 1 month, then once a day for an additional month	[213]
**Curcuminoids**	**turmeric, *Curcuma longa***	in vitro, canine tenocytes	NF-κB scleraxis TNF-α TNF-β	Calebin A suppressed inflammation and exhibited potential as preventive and therapeutic treatment of tendinitis by suppressing down-regulation of tenomodulin and collagen I.	1–10 µM of calebin A	[36]
		in vitro, human tenocytes	NF-κB IL-1βPI3K/p85/Akt MMPs COX-2 caspase-3 Bax/Bcl-2	Curcumin inhibited inflammation and apoptosis and showed potential for treatment of tendon inflammation.	5 µM of curcumin	[10]
		in vivo, diabetic rats	ROS AGE	Curcumin reduced oxidative stress by inhibiting lipid peroxidation and prevented glycation and crosslinking of advanced glycated collagen in tail tendon and skin.	200 mg/kg of curcumin for 8 weeks	[168]
		in vivo, Sprague–Dawley rats	MDA HOPro SOD	Curcumin improved the healing quality of tendon ruptures by promoting well-organized collagen filaments and biomechanical traits.	100 mg/kg of curcumin for 14 days	[64]
		in vivo, Sprague–Dawley rats	adhesion of inflammatory products	Curcumin (as loaded nanoparticle) promoted the healing process of Achilles tendon rupture.	1 injection containing 0.44 mg of curcumin/kg	[169]
		in vivo, Wistar albino rats	-	Curcumin showed biomechanical and histological healing (collagen I and III) promotion after surgically treated Achilles tendon ruptures.	200 mg/kg of curcumin for 28 days	[170]
		in vivo, rats	AP	Curcumin prevented tendon calcification and improved tendon regeneration by tendon stem/progenitor cells.	3 μg of curcumin every 3 days for up to 4 weeks	[131]
		in vivo, Sprague–Dawley rats	ROS IL-1β TNF-α MMPs	Curcumin showed anti-oxidative and anti-inflammatory properties as part of Cur&Mg-QCS/PF hydrogel application.	1 injection with 50 µL of hydrogel	[146]
		in vivo, diabetic rats	AGE HOPro	Curcumin’s metabolite tetrahydrocurcumin reduced accumulation and crosslinking of advanced glycated collagen.	80 mg/kg of tetrahydrocurcumin for 45 days	[172]
		in vivo, tendinopathy patients	-	*Curcuma longa* and *Boswellia serrata* extracts alleviated the symptoms (pain and functional limitation) of patients with tendon disease as combinational therapy.	2 tablets twice a day for 1 month	[65]
		in vivo, rotator cuff tendinopathy, Achilles tendinopathy, and lateral epicondylitis patients	-	*Curcuma longa*, *Boswellia serrata* and bromelain extracts (with methyl-sulfonyl-methane, hydrolysed collagen I and II, L-arginine, L-lysin, vitamin C, chondroitin sulfate, glucosamine, and myrrh as Tendisulfur^®^ Forte), in combination with extracorporeal shock wave therapy, accelerated pain relief and remarkably reduced NSAID intake of patients.	2 daily tablets of Tendisulfur^®^ Forte for 1 month, then once a day for an additional month	[213]
		in vivo, supraspinatus injury patients	-	*Curcuma longa* and *Boswellia serrata* extracts (as Tendisulfur^®^) reduced pain after arthroscopic supraspinatus tendon repair compared to placebo treatment.	2 daily sachets of Tendisulfur^®^ for 15 days and 1 daily sachet for the next 45 days	[212]
**EGCG (epigallocatechin gallate)**	**green tea extracts**	in vitro, human tendon-derived fibroblasts	IL-1β MMPs p54/JNK/ SAPK collagenases/ gelatinases/ aggrecanases	Green tea’s epigallocatechin gallate targeted extracellular matrix breakdown.	2.5–25 µM of epigallocatechin gallate	[148]
		in vivo, diabetic rats	AGE	Green tea extract reduced collagen glycation and crosslinking in the tail tendon.	300 mg/kg of green tea extract for 4 weeks	[145]
		in vivo, Wistar rats	MMPs HOPro	Green tea promoted the synthesis of ECM components and glycosaminoglycans, and thus the recovery process after Achilles tendinitis in combination with a glycin diet.	700 mg/kg of green tea extract for 21 days	[177]
		in vivo, Wistar rats	IL-1β MMPs	Green tea modulated inflammatory action and promoted synthesis of recovery elements after Achilles tendinitis, in combination with a glycin diet.	700 mg/kg of green tea extract for 7 days	[132]
		in vivo, C57BL/6 mice	ROS	Green tea extract slowed collagen aging by inhibiting crosslinking.	21.2 mL (young mice) and 27.2 mL (adult mice) of green tea extract for 14 days	[176]
**Echinaceae angustifolia extracts**	***Echinaceae angustifolia***	in vivo, carpal tunnel syndrome patients	-	*Echinaceae angustifolia* extract (as a dietary supplement mainly composed of alpha lipoic acid and conjugated linoleic acid) showed significant improvement in pain, symptoms, and functionality.	2 capsules containing 250 mg of echinacea extract for 40 days, then 1 capsule for 80 days	[216]
**Flavones/Flavonoids**	**celery, parsley, red peppers, chamomile, mint and gingko bilboa,** **citrus fruit**	in vitro, rat tenofibroblast	ROS ERK1/2 JNK	Flavonoid anthocyanin acted as an anti-apoptotic and showed the therapeutic potential of rotator cuff tendon.	10–200 µg/mL of anthocyanins	[133]
		in vitro, tendon stem cells	caspase-3	Flavonoid eriocitrin inhibited apoptosis and scar formation (biglycan, fibronectin, COMP) and improved woundhealing by stimulating proliferation and migration of tendon stem cells.	25–75 of µM eriocitrin	[147]
		in vivo, Sprague–Dawley rats	-	Flavone genistein protected ovariectomy-induced collagen reduction in Achilles tendon.	300 mg/kgof genistein for 6 weeks	[184]
		in vivo, Sprague–Dawley rats	Pcna Timp1	Flavone genistein enhanced tendon function at an estrogen-deficit through the modulation of tenomodulin.	6 mg/kg of genistein for 6 weeks	[185]
		in vivo, Sprague–Dawley rats	AP CD31 VEGF	Flavonoid icariin supported healing and angiogenesis after rotator cuff reconstruction through promoting collagen I/II.	0.125 mg/g of icariin for 2 and 4 weeks	[189]
		in vivo, Wistar rats	ROS MDA SOD GPX	Flavonoid quercetin prevented the adhesion of tendon tissue.	50–100 mg/kg for 4 weeks	[195]
		in vivo, Sprague–Dawley rats	MMPs ICAM-1	Flavonoid quercetin prevented collagenase-induced tendon damage at Achilles tendinopathy.	25–50 mg/kg for 7 days	[149]
		in vivo, rats	-	Flavonoid quercetin, kaempferol, and isorhamnetin (*Hippophae rhamnoides’* flavones) improved fibre alignment, collagen deposition, healing, and recovery after patellar tendon injury.	1 injection with 0.1 mg of *Hippophae rhamnoides’* flavones	[144]
**Resveratrol**	**red grapes,** ***Vitis vinifera***	in vitro, human tenocytes	NF-κB p53 Sirt-1 IL-1β COX-2 MMPs Akt/scleraxis Bax/caspase-3	Resveratrol regulated tenocytes homeostatic and inhibited inflammation of cascades and apoptosis.	5 µM of resveratrol	[62]
		in vitro, human tenocytes	Sirt-1	Resveratrol averted dexamethasone-induced senescence despite glucocorticoid treatment.	30 µM of resveratrol	[207]
		in vitro, human tenocytes	NF-κB PI3K IL-1β scleraxis	Resveratrol inhibited inflammation cascades; prevented apoptosis; and promoted collagen I, collagen III, and tenomodulin expression.	0.1–20 µM of resveratrol	[14]
		in vitro, Wistar rat tail tendon	ROS	Resveratrol’s derivate polydatin protected from advanced glycation as an anti-oxidant property.	50–500 µg of polydatin	[209]
		in vivo, Sprague–Dawley rats	-	Resveratrol promoted the collagens and the healing process of Achilles tendinopathy, despite diabetic condition.	10 mg/kg of resveratrol for 14 days	[61]

## 5. Anti-Inflammatory Effect of Nutraceuticals on Tendinitis in Clinical Trials

Currently, clinical trials investigating the effect of natural compounds on tendinitis in patients are rare. The nutraceuticals that have been studied in clinical trials in the context of tendinitis are Boswellia acid (BA) and curcumin, as concomitant agents to BA, as well as *E. angustifolia* and pineapple extracts.

Boswellia acid is the active ingredient derived from the *Boswellia serrata* tree and is found in its gum resin. BA has been used in the treatment of chronic inflammatory diseases, including bowel disease, asthma, and musculoskeletal disorders, in traditional Indian and African countries for centuries, and is known to have anti-inflammatory properties and to promote tissue regeneration [143,210,217,218]. To enhance the pharmacokinetic activity of BA, a lecithin-based system for its delivery called Casperome^®^ has been developed. Oral administration of Casperome^®^ was investigated in patients suffering from Achilles tendinitis and epicondylitis. On the one hand, the outcome of the study showed significant pain reduction of patients, and, on the other hand, tendon functionality of the injured area was strongly increased when Casperome^®^ was supplemented. Simultaneously, inflammation-linked markers in patients plasma were down-regulated, underscoring the anti-inflammatory potential of BA in tendon injury [210]. The described role of BA in the form of Casperome^®^ as a potential agent for pain relief and tissue regeneration in musculoskeletal disorders is supported by another clinical trial that examined the effects of Casperome^®^ on osteo-muscular pain triggered by inflammation of the knee tendon and joint. Here, the pain could be significantly reduced by daily oral BA supplementation together with standard therapy. However, significance of these outcomes is limited and has to be validated by larger scale investigations [211].

In another clinical trial that was conducted by Merolla et al., BA was used as a combinational therapy with the extracts of *Curcuma longa* (Tendisulfur^®^) that was orally administered concomitantly to analgesic treatment for the management of patients with supraspinatus injuries. Outcomes of Tendisulfur^®^ treatment were compared to patients, who did not receive Tendisulfur^®^ supplementation (placebo group). Within this study, Tendisulfur^®^ was found to be capable of reducing short- and mid-term post-operative pain, which in turn lead to a reduced intake of NSAIDs that is often accompanied by severe adverse effects, whereas long-term pain could not be alleviated by Tendisulfur^®^ supplementation. These results demonstrate the use of BA in combination with turmeric extracts, not only as a new strategy for post-operative pain relief, but especially as a good co-treatment strategy to reduce adverse effects accompanying the intake of NSAIDs. Altogether, *Boswellia serrata* and *Curcuma longa* extracts can be assumed to play an important role in new treatment or co-treatment strategies of tendinitis and its prevention [212]. This has also been shown in a clinical trial carried out by Henrotin et al., in which the combination of *Boswellia serrata* and *Curcuma longa*, in the form of pills, was used to analyze their effects on patients suffering from tendinopathies in a one-month trial. As described in the previous trial, this study’s results clearly showed pain and symptom relief, as well as the alleviation of the functional limitation of tendon, whereas the administration was proven to be safe during the treatment period, further suggesting these nutraceuticals as potential agents in tendon treatment or as concomitant therapy, which comes with no or less side effects [65].

In another study that was conducted by the group of Notarnicola et al., pineapple extract bromelain, together with other dietary supplements, including arginine, collagen vitamin c, methyl-sulfonyl-methane, and Vinitrox™, was supplemented by patients suffering from Achilles tendinopathy, in combination with extracorporeal shock wave therapy (ESWT), to analyze pain reduction and joint function. In the control group, patients received ESWT without dietary supplement intake, i.e., the placebo. For clinical and functional evaluation, the visual analogue scale and the ankle–hindfoot scale, as well as the Roles and Maudsley scores, were used. The results showed pain and functional improvement in the experimental group receiving dietary supplements together with ESWT, indicating that bromelain, together with concomitant dietary components, can synergistically increase the efficiency of ESWT, in comparison to the placebo group. However, the general group size of patients was limited; thus, further studies using larger sample sizes are necessary to validate these outcomes [214].

A similar diet consisting of bromelain, arginine-L-ketoglutarate, methyl-sulfonate-methane, and hydrolysed collagen I was administered to patients with a large posterior-superior rotator cuff tear in a clinical trial by Gumina et al. for 3 months [215]. For reference, another group of patients was treated without dietary supplements. Outcomes were analyzed using a pre- and post-operative constant score, whereby repair integrity was evaluated by MRI via Sugaya’s classification. In addition, results of pre- and post-operative simple shoulder tests were considered. Significant differences between the experimental group and the placebo group were mainly found in pain that was reduced with supplement administration and in integrity repair that was significantly improved in the same group. These results suggest bromelain in combinational administration for pain relief and better repair integrity in post-operative rotator cuff tendinopathy. The short follow-up period of this study and the small sample size demonstrate the limitations of these outcomes [215].

Moreover, in another clinical trial by Notarnicola et al., *Echinacea angustifolia* extracts were dietary-supplemented, combined with alpha lipoic acid and conjugated linoleic acid, which were used for the treatment strategy of carpal tunnel syndrome patients and compared to ESWT [216]. *Echinacea angustifolia* is known to exhibit strong antimicrobial, anti-inflammatory, and anti-oxidant properties, as well as immune-modulating activity [219,220]. These effects have previously been demonstrated to be partly exerted by modulation of major inflammatory pathways, including NF-κB and MAPK [220,221]. The study which compared the effects of *Echinacea angustifolia* containing dietary supplementation to ESWT treatment of carpal tunnel patients showed that both strategies revealed improvements in pain and functionality in a comparable manner, indicating the potential of nutraceuticals to be embedded in future treatment strategies, since they represent a comfortable method for the management of carpal tunnel syndrome and other tendinopathies [216].

Nutraceutical diets consisting of natural compounds *Curcuma longa*, *Boswellia serrata*, bromelain extracts and methyl sulfonyl methane, hydrolyzed collagen I and II, L-arginine, L-lysin, vitamin C, chondroitin sulfate, glucosamine, and myrrh (Tendisulfur^®^ Forte) were also analyzed in combination with ESWT in various tendinopathies, including lateral epicondylitis, Achilles, and rotator cuff tendinopathy in a clinical trial of Vitali et al. compared to patients undergoing ESWT without supplementary diet. The study’s outcome supports results of similar studies, revealing faster pain relief in tendinitis patients by nutraceutical supplementation due to the suppression of inflammation, thus leading to reduced NSAID consumption (Table 1) [213].

Overall, clinical trials that have been carried out for better knowledge of the pharmaceutic activity of nutraceuticals in inflammation-triggered tendinitis showed promising outcomes of reduced pain and down-regulated inflammatory markers in patients, leading to reduced NSAID intake and functional improvement. However, the few clinical trials that are found in the context of nutraceuticals and tendinitis focus on *Boswellia serrata* and *Curcuma longa*, as well as pineapple extract bromelain in combination with other dietary components, although many more natural compounds have been found to act as anti-inflammatory agents in pre-clinical studies [65,211,212,214,215]. Therefore, further clinical trials are urgently needed for validation of pre-clinical research of plant-derived tendinitis therapy in patients, and available results of clinical trials have to be verified in further studies with larger sample sizes. Nevertheless, existing data encourage further investigation on nutraceuticals as anti-inflammatory, tendon-protective, and pain-reducing agents.

## 6. Discussion and Perspectives

Tendon pathology is a common disease, accounting for around 30% of medical consultations because of musculoskeletal issues and pain [170]. Reasons underlying tendinitis pathologies are various, ranging from tendon rupture and mechanical overuse to a poor lifestyle and diet, as well as insufficient movement; however, in many cases, tendinitis is associated with and occurs in the most common inflammatory local and systemic diseases, such as rheumatoid arthritis, osteoarthritis, or spondyloarthritis [7,8,222,223,224]. These disorders are often accompanied by swelling, unbearable pain of tendons, and severe limitation of joint mobility and functionality [223,225,226]. Current modern therapy for tendinitis is aimed at controlling the symptoms of the disease, mainly using NSAIDs and glucocorticoids [3,20]. However, these anti-inflammatory drugs only relieve pain and swelling in tendons, but simultaneously cause severe side effects on many organs when taken long-term, such as gastrointestinal, cardiovascular, hepatic, renal, cerebral, and pulmonary systems, and are also very expensive [22,25]. Moreover, their administration has even been found to be counterproductive in some cases, because of showing inhibitory effects on proteoglycan and collagen synthesis, as well as on tendon cell proliferation, which is crucial for the regeneration and healing of tendons [29,30,208].

For these reasons, treatment alternatives that act as effective and safe drugs, thus relieving tendinitis symptoms or at least delaying the progression of the disease, are necessary. Nutraceuticals and their active ingredients can interact as multi-targeting molecules in the cells of all tissues and are able to modulate the major mediators that are assumed to cause dysregulation of inflammatory pathways which are associated with severe chronic diseases, as shown in the context of tendon pathologies and tendinitis [10,33,36,170]. Simultaneously, different polyphenols are known to help strengthen and stabilize the immune response of the human organism, thus offering great potential for the prevention and prophylactic treatment of chronic diseases [16]. Moreover, in the administration of tested natural compounds, no toxic effects or side effects were observed so far as it is assessable, fulfilling another requirement that is relevant for the sustainable management of inflammatory diseases such as tendinitis. However, currently available evidence is limited and does not draw a definite conclusion on that. Moreover, most of the studies that have been carried out in the context of nutraceuticals and tendinitis comprise pre-clinical studies, and more clinical research considering long-term efficacy and safety, besides pharmacological activity of plant-derived nutraceuticals, is still needed. Furthermore, transferring in vitro to in vivo study models bears further challenges that necessarily should be considered, such as relatively high concentrations of polyphenolic agents in vitro, which are not inevitably appropriate for in vivo use, highlighting the importance of clinical trials for further evidence. In addition, with regard to the bioavailability of plant-derived compounds as nutraceuticals and their actual treatment efficiency in practice, further clinical studies are needed for better examination and, if necessary, improvements in their pharmacologic effects. Moreover, larger-scale studies and further extensive research can help to harness the potential of nutraceuticals and to find new ways to improve the treatment of patients with serious diseases such as tendinitis.

## 7. Conclusions

Altogether, plant-derived nutraceuticals offer a promising potential treatment option for inflammation-related diseases, such as tendinitis, which concomitantly act as great drivers of anabolic processes in tendon tissue, by modulating inflammatory mediators in a safe and efficient way. In addition, these effects, together with their immuno-beneficial characteristics, make them suitable agents in tendinitis prevention or co-treatment. Nevertheless, further extensive research covering pre-clinical as well as clinical trials is necessary to overcome current limitations and to validate current findings and future possibilities.

## Data Availability

All data are available in the manuscript.

## Abbreviations

AGE	advanced glycation end-products
Akt	serine/threonine kinase B
AP	alkaline phosphatase
ASU	avocado and soybean unsaponifiables
BA	Boswellia acid
COPD	chronic destructive pulmonary disease
COX	cyclooxygenase
CS	chondroitin sulfate
ECM	extracellular matrix
EGCG	epigallochatechin-3-gallate
ESWT	extracorporeal shock wave therapy
FOXO1	forkhead box protein O1
GLU	glucosamine
GPCR	G-protein-coupled receptor
GPX	glutathione peroxidase
GSK3β	glycogen synthase kinase 3 beta
HOPro	hydroxyproline
IBD	inflammatory bowel disease
IκBα	NF-κB inhibitor alpha
IKK	IκB kinase
IL	interleukin
iNOS	inducible nitric oxide synthase
JAK	Janus kinase
MAPK	mitogen-activated protein kinase
MAPKK	MAPK kinase
MAPKKK	MAPKK kinase
MDA	malondialdehyde
MMP	matrix metalloproteinase
mTOR	mammalian target of rapamycin
NF-κB	nuclear factor ‘kappa-light-chain-enhancer’ of activated B-cells
NSAIDs	non-steroidal anti-inflammatory drugs
OA	osteoarthritis
PAF	platelet activating factor
Pcna	proliferating cell nuclear antigen
PGE2	prostaglandin E2
PI3K	phosphoinositide-3-kinase
PIP3	phosphatidylinositol 3,4,5-triphosphate
RA	rheumatoid arthritis
RANKL	receptor activator of NF-κB ligand
ROS	reactive oxygen species
SOD	superoxide dismutase
STAT3	signal transducer and activator of transcription 3
Timp1	tissue inhibitor of MMP1
TNF	tumor necrosis factor
TLR	toll-like receptor
VEGF	vascular endothelial growth factor

## References

[1] Andarawis-Puri N., Flatow E.L., Soslowsky L.J. (2015). Tendon basic science: Development, repair, regeneration, and healing. J. Orthop. Res..

[2] Millar N.L., Silbernagel K.G., Thorborg K., Kirwan P.D., Galatz L.M., Abrams G.D., Murrell G.A.C., McInnes I.B., Rodeo S.A. (2021). Tendinopathy. Nat. Rev. Dis. Prim..

[3] Childress M.A., Beutler A. (2013). Management of chronic tendon injuries. Am. Fam. Physician.

[4] D’Addona A., Maffulli N., Formisano S., Rosa D. (2017). Inflammation in tendinopathy. Surg. J. R. Coll. Surg. Edinb. Irel..

[5] Sendzik J., Shakibaei M., Schäfer-Korting M., Lode H., Stahlmann R. (2010). Synergistic effects of dexamethasone and quinolones on human-derived tendon cells. Int. J. Antimicrob. Agents.

[6] Stephenson A.L., Wu W., Cortes D., Rochon P.A. (2013). Tendon Injury and Fluoroquinolone Use: A Systematic Review. Drug Saf..

[7] Rolf C., Movin T. (1997). Etiology, histopathology, and outcome of surgery in achillodynia. Foot Ankle Int..

[8] Dakin S.G., Newton J., Martinez F.O., Hedley R., Gwilym S., Jones N., Reid H.A.B., Wood S., Wells G., Appleton L. (2018). Chronic inflammation is a feature of Achilles tendinopathy and rupture. Br. J. Sports Med..

[9] Zhong J., Shi G. (2019). Editorial: Regulation of Inflammation in Chronic Disease. Front. Immunol..

[10] Buhrmann C., Mobasheri A., Busch F., Aldinger C., Stahlmann R., Montaseri A., Shakibaei M. (2011). Curcumin modulates nuclear factor kappaB (NF-kappaB)-mediated inflammation in human tenocytes in vitro: Role of the phosphatidylinositol 3-kinase/Akt pathway. J. Biol. Chem..

[11] Lawrence T. (2009). The nuclear factor NF-kappaB pathway in inflammation. Cold Spring Harb. Perspect. Biol..

[12] Mitchell J.P., Carmody R.J. (2018). NF-κB and the Transcriptional Control of Inflammation. Int. Rev. Cell Mol. Biol..

[13] Hoesel B., Schmid J.A. (2013). The complexity of NF-κB signaling in inflammation and cancer. Mol. Cancer.

[14] Busch F., Mobasheri A., Shayan P., Lueders C., Stahlmann R., Shakibaei M. (2012). Resveratrol modulates interleukin-1β-induced phosphatidylinositol 3-kinase and nuclear factor κB signaling pathways in human tenocytes. J. Biol. Chem..

[15] Buhrmann C., Shayan P., Banik K., Kunnumakkara A.B., Kubatka P., Koklesova L., Shakibaei M. (2020). Targeting NF-κB Signaling by Calebin A, a Compound of Turmeric, in Multicellular Tumor Microenvironment: Potential Role of Apoptosis Induction in CRC Cells. Biomedicines.

[16] Kunnumakkara A.B., Shabnam B., Girisa S., Harsha C., Banik K., Devi T.B., Choudhury R., Sahu H., Parama D., Sailo B.L. (2020). Inflammation, NF-κB, and Chronic Diseases: How are They Linked?. Crit. Rev. Immunol..

[17] Aggarwal B.B., Sung B. (2011). NF-κB in cancer: A matter of life and death. Cancer Discov..

[18] Gupta S.C., Kim J.H., Kannappan R., Reuter S., Dougherty P.M., Aggarwal B.B. (2011). Role of nuclear factor κB-mediated inflammatory pathways in cancer-related symptoms and their regulation by nutritional agents. Exp. Biol. Med..

[19] Bernard-Beaubois K., Hecquet C., Houcine O., Hayem G., Adolphe M. (1997). Culture and characterization of juvenile rabbit tenocytes. Cell Biol. Toxicol..

[20] Chianca V., Albano D., Messina C., Midiri F., Mauri G., Aliprandi A., Catapano M., Pescatori L.C., Monaco C.G., Gitto S. (2018). Rotator cuff calcific tendinopathy: From diagnosis to treatment. Acta Bio-Med. Atenei Parm..

[21] Bacchi S., Palumbo P., Sponta A., Coppolino M.F. (2012). Clinical pharmacology of non-steroidal anti-inflammatory drugs: A review. Anti-Inflamm. Anti-Allergy Agents Med. Chem..

[22] Bindu S., Mazumder S., Bandyopadhyay U. (2020). Non-steroidal anti-inflammatory drugs (NSAIDs) and organ damage: A current perspective. Biochem. Pharmacol..

[23] Cain D.W., Cidlowski J.A. (2017). Immune regulation by glucocorticoids. Nat. Reviews. Immunol..

[24] Vandewalle J., Luypaert A., De Bosscher K., Libert C. (2018). Therapeutic Mechanisms of Glucocorticoids. Trends Endocrinol. Metab. TEM.

[25] Süleyman H., Demircan B., Karagöz Y. (2007). Anti-inflammatory and side effects of cyclooxygenase inhibitors. Pharmacol. Rep. PR.

[26] Grosser T., Ricciotti E., FitzGerald G.A. (2017). The Cardiovascular Pharmacology of Nonsteroidal Anti-Inflammatory Drugs. Trends Pharmacol. Sci..

[27] Riley G.P., Curry V., DeGroot J., van El B., Verzijl N., Hazleman B.L., Bank R.A. (2002). Matrix metalloproteinase activities and their relationship with collagen remodelling in tendon pathology. Matrix Biol. J. Int. Soc. Matrix Biol..

[28] Riley G.P., Cox M., Harrall R.L., Clements S., Hazleman B.L. (2001). Inhibition of tendon cell proliferation and matrix glycosaminoglycan synthesis by non-steroidal anti-inflammatory drugs in vitro. J. Hand Surg..

[29] Tillander B., Franzén L.E., Karlsson M.H., Norlin R. (1999). Effect of steroid injections on the rotator cuff: An experimental study in rats. J. Shoulder Elb. Surg..

[30] Akpinar S., Hersekli M.A., Demirors H., Tandogan R.N., Kayaselcuk F. (2002). Effects of methylprednisolone and betamethasone injections on the rotator cuff: An experimental study in rats. Adv. Ther..

[31] Cheng A.L., Hsu C.H., Lin J.K., Hsu M.M., Ho Y.F., Shen T.S., Ko J.Y., Lin J.T., Lin B.R., Ming-Shiang W. (2001). Phase I clinical trial of curcumin, a chemopreventive agent, in patients with high-risk or pre-malignant lesions. Anticancer. Res..

[32] Duvoix A., Blasius R., Delhalle S., Schnekenburger M., Morceau F., Henry E., Dicato M., Diederich M. (2005). Chemopreventive and therapeutic effects of curcumin. Cancer Lett..

[33] Das S., Das D.K. (2007). Anti-inflammatory responses of resveratrol. Inflamm. Allergy Drug Targets.

[34] Aggarwal B.B., Yuan W., Li S., Gupta S.C. (2013). Curcumin-free turmeric exhibits anti-inflammatory and anticancer activities: Identification of novel components of turmeric. Mol. Nutr. Food Res..

[35] He Y., Yue Y., Zheng X., Zhang K., Chen S., Du Z. (2015). Curcumin, inflammation, and chronic diseases: How are they linked?. Molecules.

[36] Mueller A.L., Brockmueller A., Kunnumakkara A.B., Shakibaei M. (2022). Calebin A, a Compound of Turmeric, Down-Regulates Inflammation in Tenocytes by NF-κB/Scleraxis Signaling. Int. J. Mol. Sci..

[37] Buhrmann C., Shayan P., Brockmueller A., Shakibaei M. (2020). Resveratrol Suppresses Cross-Talk between Colorectal Cancer Cells and Stromal Cells in Multicellular Tumor Microenvironment: A Bridge between In Vitro and In Vivo Tumor Microenvironment Study. Molecules.

[38] Miyata Y., Shida Y., Hakariya T., Sakai H. (2019). Anti-Cancer Effects of Green Tea Polyphenols Against Prostate Cancer. Molecules.

[39] Maiuolo J., Gliozzi M., Carresi C., Musolino V., Oppedisano F., Scarano F., Nucera S., Scicchitano M., Bosco F., Macri R. (2021). Nutraceuticals and Cancer: Potential for Natural Polyphenols. Nutrients.

[40] Sajadimajd S., Bahramsoltani R., Iranpanah A., Kumar Patra J., Das G., Gouda S., Rahimi R., Rezaeiamiri E., Cao H., Giampieri F. (2020). Advances on Natural Polyphenols as Anticancer Agents for Skin Cancer. Pharmacol. Res..

[41] McGrattan A.M., McGuinness B., McKinley M.C., Kee F., Passmore P., Woodside J.V., McEvoy C.T. (2019). Diet and Inflammation in Cognitive Ageing and Alzheimer’s Disease. Curr. Nutr. Rep..

[42] Moussa C., Hebron M., Huang X., Ahn J., Rissman R.A., Aisen P.S., Turner R.S. (2017). Resveratrol regulates neuro-inflammation and induces adaptive immunity in Alzheimer’s disease. J. Neuroinflam..

[43] Rehman M.U., Wali A.F., Ahmad A., Shakeel S., Rasool S., Ali R., Rashid S.M., Madkhali H., Ganaie M.A., Khan R. (2019). Neuroprotective Strategies for Neurological Disorders by Natural Products: An update. Curr. Neuropharmacol..

[44] Rahman I. (2006). Antioxidant therapies in COPD. Int. J. Chronic Obstr. Pulm. Dis..

[45] Wang X.L., Li T., Li J.H., Miao S.Y., Xiao X.Z. (2017). The Effects of Resveratrol on Inflammation and Oxidative Stress in a Rat Model of Chronic Obstructive Pulmonary Disease. Molecules.

[46] Van Iersel L.E.J., Beijers R., Gosker H.R., Schols A. (2022). Nutrition as a modifiable factor in the onset and progression of pulmonary function impairment in COPD: A systematic review. Nutr. Rev..

[47] Biswas S., Hwang J.W., Kirkham P.A., Rahman I. (2013). Pharmacological and dietary antioxidant therapies for chronic obstructive pulmonary disease. Curr. Med. Chem..

[48] Nardo V.D., Gianfaldoni S., Tchernev G., Wollina U., Barygina V., Lotti J., Daaboul F., Lotti T. (2018). Use of Curcumin in Psoriasis. Open Access Maced. J. Med. Sci..

[49] Maioli E., Valacchi G. (2010). Rottlerin: Bases for a possible usage in psoriasis. Curr. Drug Metab..

[50] Dimitris D., Ekaterina-Michaela T., Christina K., Ioannis S., Ioanna S.K., Aggeliki L., Sophia H., Michael R., Helen S. (2020). Melissa officinalis ssp. altissima extracts: A therapeutic approach targeting psoriasis in mice. J. Ethnopharmacol..

[51] Yamagata K. (2019). Polyphenols Regulate Endothelial Functions and Reduce the Risk of Cardiovascular Disease. Curr. Pharm. Des..

[52] Khan N., Khymenets O., Urpí-Sardà M., Tulipani S., Garcia-Aloy M., Monagas M., Mora-Cubillos X., Llorach R., Andres-Lacueva C. (2014). Cocoa polyphenols and inflammatory markers of cardiovascular disease. Nutrients.

[53] Vahdat-Lasemi F., Aghaee-Bakhtiari S.H., Tasbandi A., Jaafari M.R., Sahebkar A. (2021). Targeting interleukin-β by plant-derived natural products: Implications for the treatment of atherosclerotic cardiovascular disease. Phytother. Res. PTR.

[54] Buhrmann C., Brockmueller A., Mueller A.L., Shayan P., Shakibaei M. (2021). Curcumin Attenuates Environment-Derived Osteoarthritis by Sox9/NF-kB Signaling Axis. Int. J. Mol. Sci..

[55] Gupta S.C., Kunnumakkara A.B., Aggarwal S., Aggarwal B.B. (2018). Inflammation, a Double-Edge Sword for Cancer and Other Age-Related Diseases. Front. Immunol..

[56] Kunnumakkara A.B., Sailo B.L., Banik K., Harsha C., Prasad S., Gupta S.C., Bharti A.C., Aggarwal B.B. (2018). Chronic diseases, inflammation, and spices: How are they linked?. J. Transl. Med..

[57] Athanassiou P., Athanassiou L., Kostoglou-Athanassiou I. (2020). Nutritional Pearls: Diet and Rheumatoid Arthritis. Mediterr. J. Rheumatol..

[58] Bellavia D., Caradonna F., Dimarco E., Costa V., Carina V., De Luca A., Raimondi L., Fini M., Gentile C., Giavaresi G. (2021). Non-flavonoid polyphenols in osteoporosis: Preclinical evidence. Trends Endocrinol. Metab. TEM.

[59] Jiang Y., Luo W., Wang B., Wang X., Gong P., Xiong Y. (2020). Resveratrol promotes osteogenesis via activating SIRT1/FoxO1 pathway in osteoporosis mice. Life Sci..

[60] Li X., Lin F., Wu Y., Liu N., Wang J., Chen R., Lu Z. (2019). Resveratrol attenuates inflammation environment-induced nucleus pulposus cell senescence in vitro. Biosci. Rep..

[61] Zeytin K., Ciloğlu N.S., Ateş F., Vardar Aker F., Ercan F. (2014). The effects of resveratrol on tendon healing of diabetic rats. Acta Orthop. Et Traumatol. Turc..

[62] Busch F., Mobasheri A., Shayan P., Stahlmann R., Shakibaei M. (2012). Sirt-1 is required for the inhibition of apoptosis and inflammatory responses in human tenocytes. J. Biol. Chem..

[63] Madhan B., Muralidharan C., Jayakumar R. (2002). Study on the stabilisation of collagen with vegetable tannins in the presence of acrylic polymer. Biomaterials.

[64] Jiang D., Gao P., Lin H., Geng H. (2016). Curcumin improves tendon healing in rats: A histological, biochemical, and functional evaluation. Connect. Tissue Res..

[65] Henrotin Y., Dierckxsens Y., Delisse G., Seidel L., Albert A. (2021). Curcuminoids and Boswellia serrata extracts combination decreases tendinopathy symptoms: Findings from an open-label post-observational study. Curr. Med. Res. Opin..

[66] Schindler J.F., Monahan J.B., Smith W.G. (2007). p38 pathway kinases as anti-inflammatory drug targets. J. Dent. Res..

[67] Yeung Y.T., Aziz F., Guerrero-Castilla A., Arguelles S. (2018). Signaling Pathways in Inflammation and Anti-inflammatory Therapies. Curr. Pharm. Des..

[68] Libby P. (2007). Inflammatory mechanisms: The molecular basis of inflammation and disease. Nutr. Rev..

[69] Yong H.Y., Koh M.S., Moon A. (2009). The p38 MAPK inhibitors for the treatment of inflammatory diseases and cancer. Expert Opin. Investig. Drugs.

[70] Coulthard L.R., White D.E., Jones D.L., McDermott M.F., Burchill S.A. (2009). p38(MAPK): Stress responses from molecular mechanisms to therapeutics. Trends Mol. Med..

[71] Shakibaei M., Buhrmann C., Mobasheri A. (2011). Resveratrol-mediated SIRT-1 interactions with p300 modulate receptor activator of NF-kappaB ligand (RANKL) activation of NF-kappaB signaling and inhibit osteoclastogenesis in bone-derived cells. J. Biol. Chem..

[72] Deeks E.D. (2018). Denosumab: A Review in Postmenopausal Osteoporosis. Drugs Aging.

[73] Udagawa N., Koide M., Nakamura M., Nakamichi Y., Yamashita T., Uehara S., Kobayashi Y., Furuya Y., Yasuda H., Fukuda C. (2021). Osteoclast differentiation by RANKL and OPG signaling pathways. J. Bone Miner. Metab..

[74] Johnson D.E., O’Keefe R.A., Grandis J.R. (2018). Targeting the IL-6/JAK/STAT3 signalling axis in cancer. Nat. Rev. Clin. Oncol..

[75] Kumari N., Dwarakanath B.S., Das A., Bhatt A.N. (2016). Role of interleukin-6 in cancer progression and therapeutic resistance. Tumour Biol. J. Int. Soc. Oncodev. Biol. Med..

[76] Bournazou E., Bromberg J. (2013). Targeting the tumor microenvironment: JAK-STAT3 signaling. Jak-Stat.

[77] Yu H., Pardoll D., Jove R. (2009). STATs in cancer inflammation and immunity: A leading role for STAT3. Nat. Rev. Cancer.

[78] Hawkins P.T., Stephens L.R. (2015). PI3K signalling in inflammation. Biochim. Biophys. Acta.

[79] Lien E.C., Dibble C.C., Toker A. (2017). PI3K signaling in cancer: Beyond AKT. Curr. Opin. Cell Biol..

[80] Pompura S.L., Dominguez-Villar M. (2018). The PI3K/AKT signaling pathway in regulatory T-cell development, stability, and function. J. Leukoc. Biol..

[81] Song M., Bode A.M., Dong Z., Lee M.H. (2019). AKT as a Therapeutic Target for Cancer. Cancer Res..

[82] Nguyen D.P., Li J., Yadav S.S., Tewari A.K. (2014). Recent insights into NF-κB signalling pathways and the link between inflammation and prostate cancer. BJU Int..

[83] Shishodia S., Aggarwal B.B. (2004). Nuclear factor-kappaB: A friend or a foe in cancer?. Biochem. Pharmacol..

[84] Karin M., Ben-Neriah Y. (2000). Phosphorylation meets ubiquitination: The control of NF-[kappa]B activity. Annu. Rev. Immunol..

[85] Oeckinghaus A., Hayden M.S., Ghosh S. (2011). Crosstalk in NF-κB signaling pathways. Nat. Immunol..

[86] Mitchell S., Vargas J., Hoffmann A. (2016). Signaling via the NFκB system. Wiley Interdiscip. Rev. Syst. Biol. Med..

[87] Pahl H.L. (1999). Activators and target genes of Rel/NF-kappaB transcription factors. Oncogene.

[88] Fan Y., Mao R., Yang J. (2013). NF-κB and STAT3 signaling pathways collaboratively link inflammation to cancer. Protein Cell.

[89] Sun S.C. (2017). The non-canonical NF-κB pathway in immunity and inflammation. Nat. Rev. Immunol..

[90] Li L., Aggarwal B.B., Shishodia S., Abbruzzese J., Kurzrock R. (2004). Nuclear factor-kappaB and IkappaB kinase are constitutively active in human pancreatic cells, and their down-regulation by curcumin (diferuloylmethane) is associated with the suppression of proliferation and the induction of apoptosis. Cancer.

[91] Kotha R.R., Luthria D.L. (2019). Curcumin: Biological, Pharmaceutical, Nutraceutical, and Analytical Aspects. Molecules.

[92] Ansari M.Y., Ahmad N., Haqqi T.M. (2020). Oxidative stress and inflammation in osteoarthritis pathogenesis: Role of polyphenols. Biomed. Pharmacother. Biomed. Pharmacother..

[93] Behl T., Upadhyay T., Singh S., Chigurupati S., Alsubayiel A.M., Mani V., Vargas-De-La-Cruz C., Uivarosan D., Bustea C., Sava C. (2021). Polyphenols Targeting MAPK Mediated Oxidative Stress and Inflammation in Rheumatoid Arthritis. Molecules.

[94] Shakoor H., Feehan J., Apostolopoulos V., Platat C., Al Dhaheri A.S., Ali H.I., Ismail L.C., Bosevski M., Stojanovska L. (2021). Immunomodulatory Effects of Dietary Polyphenols. Nutrients.

[95] Tangney C.C., Rasmussen H.E. (2013). Polyphenols, inflammation, and cardiovascular disease. Curr. Atheroscler. Rep..

[96] Niedzwiecki A., Roomi M.W., Kalinovsky T., Rath M. (2016). Anticancer Efficacy of Polyphenols and Their Combinations. Nutrients.

[97] Chaplin A., Carpéné C., Mercader J. (2018). Resveratrol, Metabolic Syndrome, and Gut Microbiota. Nutrients.

[98] Serino A., Salazar G. (2018). Protective Role of Polyphenols against Vascular Inflammation, Aging and Cardiovascular Disease. Nutrients.

[99] Khan H., Ullah H., Castilho P., Gomila A.S., D’Onofrio G., Filosa R., Wang F., Nabavi S.M., Daglia M., Silva A.S. (2020). Targeting NF-κB signaling pathway in cancer by dietary polyphenols. Crit. Rev. Food Sci. Nutr..

[100] Hazafa A., Rehman K.U., Jahan N., Jabeen Z. (2020). The Role of Polyphenol (Flavonoids) Compounds in the Treatment of Cancer Cells. Nutr. Cancer.

[101] Ren Z., Wang L., Cui J., Huoc Z., Xue J., Cui H., Mao Q., Yang R. (2013). Resveratrol inhibits NF-kB signaling through suppression of p65 and IkappaB kinase activities. Die Pharm..

[102] Buhrmann C., Popper B., Kunnumakkara A.B., Aggarwal B.B., Shakibaei M. (2019). Evidence That Calebin A, a Component of Curcuma Longa Suppresses NF-B Mediated Proliferation, Invasion and Metastasis of Human Colorectal Cancer Induced by TNF-β (Lymphotoxin). Nutrients.

[103] Seo E.J., Fischer N., Efferth T. (2018). Phytochemicals as inhibitors of NF-κB for treatment of Alzheimer’s disease. Pharmacol. Res..

[104] Nam N.H. (2006). Naturally occurring NF-kappaB inhibitors. Mini Rev. Med. Chem..

[105] Kang J., Thakali K.M., Jensen G.S., Wu X. (2015). Phenolic acids of the two major blueberry species in the US Market and their antioxidant and anti-inflammatory activities. Plant Foods Hum. Nutr..

[106] Aranaz P., Romo-Hualde A., Zabala M., Navarro-Herrera D., Ruiz de Galarreta M., Gil A.G., Martinez J.A., Milagro F.I., González-Navarro C.J. (2017). Freeze-dried strawberry and blueberry attenuates diet-induced obesity and insulin resistance in rats by inhibiting adipogenesis and lipogenesis. Food Funct..

[107] Ma L., Sun Z., Zeng Y., Luo M., Yang J. (2018). Molecular Mechanism and Health Role of Functional Ingredients in Blueberry for Chronic Disease in Human Beings. Int. J. Mol. Sci..

[108] Kuner P., Schubenel R., Hertel C. (1998). Beta-amyloid binds to p57NTR and activates NFkappaB in human neuroblastoma cells. J. Neurosci. Res..

[109] Ono K., Hasegawa K., Naiki H., Yamada M. (2004). Curcumin has potent anti-amyloidogenic effects for Alzheimer’s beta-amyloid fibrils in vitro. J. Neurosci. Res..

[110] Ghasemi F., Shafiee M., Banikazemi Z., Pourhanifeh M.H., Khanbabaei H., Shamshirian A., Amiri Moghadam S., ArefNezhad R., Sahebkar A., Avan A. (2019). Curcumin inhibits NF-kB and Wnt/β-catenin pathways in cervical cancer cells. Pathol. Res. Pract..

[111] Wang Y., Tang Q., Duan P., Yang L. (2018). Curcumin as a therapeutic agent for blocking NF-κB activation in ulcerative colitis. Immunopharmacol. Immunotoxicol..

[112] Hayakawa T., Yaguchi T., Kawakami Y. (2020). Enhanced anti-tumor effects of the PD-1 blockade combined with a highly absorptive form of curcumin targeting STAT3. Cancer Sci..

[113] Cao W., Zhang Y., Li A., Yu P., Song L., Liang J., Cao N., Gao J., Xu R., Ma Y. (2021). Curcumin reverses hepatic epithelial mesenchymal transition induced by trichloroethylene by inhibiting IL-6R/STAT3. Toxicol. Mech. Methods.

[114] Alexandrow M.G., Song L.J., Altiok S., Gray J., Haura E.B., Kumar N.B. (2012). Curcumin: A novel Stat3 pathway inhibitor for chemoprevention of lung cancer. Eur. J. Cancer Prev..

[115] Wang X., Liu M., Cai G.H., Chen Y., Shi X.C., Zhang C.C., Xia B., Xie B.C., Liu H., Zhang R.X. (2020). A Potential Nutraceutical Candidate Lactucin Inhibits Adipogenesis through Downregulation of JAK2/STAT3 Signaling Pathway-Mediated Mitotic Clonal Expansion. Cells.

[116] Jang J.H., Park C.Y., Sung E.G., Song I.H., Kim J.Y., Jung C., Sohn H.Y., Lee T.J. (2021). Lactucin induces apoptosis through reactive oxygen species-mediated BCL-2 and CFLAR(L) downregulation in Caki-1 cells. Genes Genom..

[117] Yang G., Chang C.C., Yang Y., Yuan L., Xu L., Ho C.T., Li S. (2018). Resveratrol Alleviates Rheumatoid Arthritis via Reducing ROS and Inflammation, Inhibiting MAPK Signaling Pathways, and Suppressing Angiogenesis. J. Agric. Food Chem..

[118] Hou Y., Wang K., Wan W., Cheng Y., Pu X., Ye X. (2018). Resveratrol provides neuroprotection by regulating the JAK2/STAT3/PI3K/AKT/mTOR pathway after stroke in rats. Genes Dis..

[119] Negri A., Naponelli V., Rizzi F., Bettuzzi S. (2018). Molecular Targets of Epigallocatechin-Gallate (EGCG): A Special Focus on Signal Transduction and Cancer. Nutrients.

[120] Chen T., Zhang X., Zhu G., Liu H., Chen J., Wang Y., He X. (2020). Quercetin inhibits TNF-α induced HUVECs apoptosis and inflammation via downregulating NF-kB and AP-1 signaling pathway in vitro. Medicine.

[121] Nani A., Murtaza B., Sayed Khan A., Khan N.A., Hichami A. (2021). Antioxidant and Anti-Inflammatory Potential of Polyphenols Contained in Mediterranean Diet in Obesity: Molecular Mechanisms. Molecules.

[122] Tuzcu Z., Orhan C., Sahin N., Juturu V., Sahin K. (2017). Cinnamon Polyphenol Extract Inhibits Hyperlipidemia and Inflammation by Modulation of Transcription Factors in High-Fat Diet-Fed Rats. Oxidative Med. Cell. Longev..

[123] Fraga C.G., Croft K.D., Kennedy D.O., Tomás-Barberán F.A. (2019). The effects of polyphenols and other bioactives on human health. Food Funct..

[124] Reuter S., Gupta S.C., Chaturvedi M.M., Aggarwal B.B. (2010). Oxidative stress, inflammation, and cancer: How are they linked?. Free. Radic. Biol. Med..

[125] Domazetovic V., Marcucci G., Iantomasi T., Brandi M.L., Vincenzini M.T. (2017). Oxidative stress in bone remodeling: Role of antioxidants. Clin. Cases Miner. Bone Metab..

[126] Feng K., Ge Y., Chen Z., Li X., Liu Z., Li X., Li H., Tang T., Yang F., Wang X. (2019). Curcumin Inhibits the PERK-eIF2α-CHOP Pathway through Promoting SIRT1 Expression in Oxidative Stress-induced Rat Chondrocytes and Ameliorates Osteoarthritis Progression in a Rat Model. Oxidative Med. Cell. Longev..

[127] Wang P., Ye Y., Yuan W., Tan Y., Zhang S., Meng Q. (2021). Curcumin exerts a protective effect on murine knee chondrocytes treated with IL-1β through blocking the NF-κB/HIF-2α signaling pathway. Ann. Transl. Med..

[128] Meng T., Xiao D., Muhammed A., Deng J., Chen L., He J. (2021). Anti-Inflammatory Action and Mechanisms of Resveratrol. Molecules.

[129] Ammon H.P.T. (2019). Boswellic extracts and 11-keto-ß-boswellic acids prevent type 1 and type 2 diabetes mellitus by suppressing the expression of proinflammatory cytokines. Phytomed. Int. J. Phytother. Phytopharm..

[130] Aiyegbusi A.I., Duru F.I., Anunobi C.C., Noronha C.C., Okanlawon A.O. (2011). Bromelain in the early phase of healing in acute crush Achilles tendon injury. Phytother. Res. PTR.

[131] Chen Y., Xie Y., Liu M., Hu J., Tang C., Huang J., Qin T., Chen X., Chen W., Shen W. (2019). Controlled-release curcumin attenuates progression of tendon ectopic calcification by regulating the differentiation of tendon stem/progenitor cells. Mater. Sci. Eng. C Mater. Biol. Appl..

[132] Vieira C.P., De Oliveira L.P., Da Ré Guerra F., Marcondes M.C., Pimentel E.R. (2016). Green Tea and Glycine Modulate the Activity of Metalloproteinases and Collagen in the Tendinitis of the Myotendinous Junction of the Achilles Tendon. Anat. Rec..

[133] Park H.B., Hah Y.S., Yang J.W., Nam J.B., Cho S.H., Jeong S.T. (2010). Antiapoptotic effects of anthocyanins on rotator cuff tenofibroblasts. J. Orthop. Res..

[134] Farzaei M.H., Bahramsoltani R., Abdolghaffari A.H., Sodagari H.R., Esfahani S.A., Rezaei N. (2016). A mechanistic review on plant-derived natural compounds as dietary supplements for prevention of inflammatory bowel disease. Expert Rev. Gastroenterol. Hepatol..

[135] Manach C., Scalbert A., Morand C., Rémésy C., Jiménez L. (2004). Polyphenols: Food sources and bioavailability. Am. J. Clin. Nutr..

[136] Olivares-Vicente M., Barrajon-Catalan E., Herranz-Lopez M., Segura-Carretero A., Joven J., Encinar J.A., Micol V. (2018). Plant-Derived Polyphenols in Human Health: Biological Activity, Metabolites and Putative Molecular Targets. Curr. Drug Metab..

[137] Anand P., Kunnumakkara A.B., Newman R.A., Aggarwal B.B. (2007). Bioavailability of curcumin: Problems and promises. Mol. Pharm..

[138] Di Lorenzo C., Colombo F., Biella S., Stockley C., Restani P. (2021). Polyphenols and Human Health: The Role of Bioavailability. Nutrients.

[139] Abate M., Silbernagel K.G., Siljeholm C., Di Iorio A., De Amicis D., Salini V., Werner S., Paganelli R. (2009). Pathogenesis of tendinopathies: Inflammation or degeneration?. Arthritis Res. Ther..

[140] Speed C. (2016). Inflammation in Tendon Disorders. Adv. Exp. Med. Biol..

[141] McHugh J. (2019). Targeting NF-κB in tendinopathy. Nat. Rev. Rheumatol..

[142] Sharma P., Maffulli N. (2006). Biology of tendon injury: Healing, modeling and remodeling. J. Musculoskelet. Neuronal Interact..

[143] Abdel-Tawab M., Werz O., Schubert-Zsilavecz M. (2011). Boswellia serrata: An overall assessment of in vitro, preclinical, pharmacokinetic and clinical data. Clin. Pharmacokinet..

[144] Fu S.C., Hui C.W., Li L.C., Cheuk Y.C., Qin L., Gao J., Chan K.M. (2005). Total flavones of Hippophae rhamnoides promotes early restoration of ultimate stress of healing patellar tendon in a rat model. Med. Eng. Phys..

[145] Babu P.V., Sabitha K.E., Shyamaladevi C.S. (2008). Effect of green tea extract on advanced glycation and cross-linking of tail tendon collagen in streptozotocin induced diabetic rats. Food Chem. Toxicol. Int. J. Publ. Br. Ind. Biol. Res. Assoc..

[146] Chen B., Liang Y., Zhang J., Bai L., Xu M., Han Q., Han X., Xiu J., Li M., Zhou X. (2021). Synergistic enhancement of tendon-to-bone healing via anti-inflammatory and pro-differentiation effects caused by sustained release of Mg^2+^/curcumin from injectable self-healing hydrogels. Theranostics.

[147] Shang C., Tian Z., Li G., Liu G., Zhang H. (2021). Effect of Eriocitrin on Cell Proliferation, Apoptosis, Migration, and Scar Formation-Related Genes Expression in Tendon Stem Cells. Doklady. Biochem. Biophys..

[148] Corps A.N., Curry V.A., Buttle D.J., Hazleman B.L., Riley G.P. (2004). Inhibition of interleukin-1beta-stimulated collagenase and stromelysin expression in human tendon fibroblasts by epigallocatechin gallate ester. Matrix Biol. J. Int. Soc. Matrix Biol..

[149] Semis H.S., Gur C., Ileriturk M., Kandemir F.M., Kaynar O. (2022). Evaluation of Therapeutic Effects of Quercetin Against Achilles Tendinopathy in Rats via Oxidative Stress, Inflammation, Apoptosis, Autophagy, and Metalloproteinases. Am. J. Sports Med..

[150] Davis M.E., Gumucio J.P., Sugg K.B., Bedi A., Mendias C.L. (2013). MMP inhibition as a potential method to augment the healing of skeletal muscle and tendon extracellular matrix. J. Appl. Physiol..

[151] Diniz-Fernandes T., Godoy-Santos A.L., Santos M.C., Pontin P., Pereira C.A.A., Jardim Y.J., Velosa A.P.P., Maffulli N., Teodoro W.R., Capelozzi V.L. (2018). Matrix metalloproteinase-1 (MMP-1) and (MMP-8) gene polymorphisms promote increase and remodeling of the collagen III and V in posterior tibial tendinopathy. Histol. Histopathol..

[152] Loiselle A.E., Bragdon G.A., Jacobson J.A., Hasslund S., Cortes Z.E., Schwarz E.M., Mitten D.J., Awad H.A., O’Keefe R.J. (2009). Remodeling of murine intrasynovial tendon adhesions following injury: MMP and neotendon gene expression. J. Orthop. Res..

[153] Salehi B., Rescigno A., Dettori T., Calina D., Docea A.O., Singh L., Cebeci F., Özçelik B., Bhia M., Dowlati Beirami A. (2020). Avocado-Soybean Unsaponifiables: A Panoply of Potentialities to Be Exploited. Biomolecules.

[154] Ernst E. (2003). Avocado-soybean unsaponifiables (ASU) for osteoarthritis—A systematic review. Clin. Rheumatol..

[155] Angermann P. (2005). Avocado/soybean unsaponifiables in the treatment of knee and hip osteoarthritis. Ugeskr. Laeger.

[156] Al-Afify A.S.A., El-Akabawy G., El-Sherif N.M., El-Safty F.E.A., El-Habiby M.M. (2018). Avocado soybean unsaponifiables ameliorates cartilage and subchondral bone degeneration in mono-iodoacetate-induced knee osteoarthritis in rats. Tissue Cell.

[157] Grzanna M.W., Au R.Y., Au A.Y., Rashmir A.M., Frondoza C.G. (2020). Avocado/Soybean Unsaponifiables, Glucosamine and Chondroitin Sulfate Combination Inhibits Proinflammatory COX-2 Expression and Prostaglandin E2 Production in Tendon-Derived Cells. J. Med. Food.

[158] Rathnavelu V., Alitheen N.B., Sohila S., Kanagesan S., Ramesh R. (2016). Potential role of bromelain in clinical and therapeutic applications. Biomed. Rep..

[159] Orsini R.A. (2006). Bromelain. Plast. Reconstr. Surg..

[160] Fitzhugh D.J., Shan S., Dewhirst M.W., Hale L.P. (2008). Bromelain treatment decreases neutrophil migration to sites of inflammation. Clin. Immunol..

[161] Aiyegbusi A.I., Duru F.I., Awelimobor D., Noronha C.C., Okanlawon A.O. (2010). The role of aqueous extract of pineapple fruit parts on the healing of acute crush tendon injury. Niger. Q. J. Hosp. Med..

[162] Del Rio D., Stewart A.J., Pellegrini N. (2005). A review of recent studies on malondialdehyde as toxic molecule and biological marker of oxidative stress. Nutr. Metab. Cardiovasc. Dis. NMCD.

[163] Aiyegbusi A.I., Olabiyi O.O., Duru F.I., Noronha C.C., Okanlawon A.O. (2011). A comparative study of the effects of bromelain and fresh pineapple juice on the early phase of healing in acute crush achilles tendon injury. J. Med. Food.

[164] Silvestro S., Sindona C., Bramanti P., Mazzon E. (2021). A State of the Art of Antioxidant Properties of Curcuminoids in Neurodegenerative Diseases. Int. J. Mol. Sci..

[165] Gupta S.C., Sung B., Kim J.H., Prasad S., Li S., Aggarwal B.B. (2013). Multitargeting by turmeric, the golden spice: From kitchen to clinic. Mol. Nutr. Food Res..

[166] Tyagi A.K., Prasad S., Majeed M., Aggarwal B.B. (2017). Calebin A, a novel component of turmeric, suppresses NF-κB regulated cell survival and inflammatory gene products leading to inhibition of cell growth and chemosensitization. Phytomed. Int. J. Phytother. Phytopharm..

[167] Pulido-Moran M., Moreno-Fernandez J., Ramirez-Tortosa C., Ramirez-Tortosa M. (2016). Curcumin and Health. Molecules.

[168] Sajithlal G.B., Chithra P., Chandrakasan G. (1998). Effect of curcumin on the advanced glycation and cross-linking of collagen in diabetic rats. Biochem. Pharmacol..

[169] Zhang W., Li X., Comes Franchini M., Xu K., Locatelli E., Martin R.C., Monaco I., Li Y., Cui S. (2016). Controlled release of curcumin from curcumin-loaded nanomicelles to prevent peritendinous adhesion during Achilles tendon healing in rats. Int. J. Nanomed..

[170] Güleç A., Türk Y., Aydin B.K., Erkoçak Ö.F., Safalı S., Ugurluoglu C. (2018). Effect of curcumin on tendon healing: An experimental study in a rat model of Achilles tendon injury. Int. Orthop..

[171] Fusini F., Bisicchia S., Bottegoni C., Gigante A., Zanchini F., Busilacchi A. (2016). Nutraceutical supplement in the management of tendinopathies: A systematic review. Muscles Ligaments Tendons J..

[172] Pari L., Murugan P. (2007). Influence of tetrahydrocurcumin on tail tendon collagen contents and its properties in rats with streptozotocin-nicotinamide-induced type 2 diabetes. Fundam. Clin. Pharmacol..

[173] Ohishi T., Goto S., Monira P., Isemura M., Nakamura Y. (2016). Anti-inflammatory Action of Green Tea. Anti-Inflamm. Anti-Allergy Agents Med. Chem..

[174] Legeay S., Rodier M., Fillon L., Faure S., Clere N. (2015). Epigallocatechin Gallate: A Review of Its Beneficial Properties to Prevent Metabolic Syndrome. Nutrients.

[175] Chu C., Deng J., Man Y., Qu Y. (2017). Green Tea Extracts Epigallocatechin-3-gallate for Different Treatments. BioMed Res. Int..

[176] Rutter K., Sell D.R., Fraser N., Obrenovich M., Zito M., Starke-Reed P., Monnier V.M. (2003). Green tea extract suppresses the age-related increase in collagen crosslinking and fluorescent products in C57BL/6 mice. Int. J. Vitam. Nutr. Res..

[177] Vieira C.P., Guerra Fda R., de Oliveira L.P., Almeida M.S., Marcondes M.C., Pimentell E.R. (2015). Green tea and glycine aid in the recovery of tendinitis of the Achilles tendon of rats. Connect. Tissue Res..

[178] Serafini M., Peluso I., Raguzzini A. (2010). Flavonoids as anti-inflammatory agents. Proc. Nutr. Soc..

[179] Wang K., Lv Q., Miao Y.M., Qiao S.M., Dai Y., Wei Z.F. (2018). Cardamonin, a natural flavone, alleviates inflammatory bowel disease by the inhibition of NLRP3 inflammasome activation via an AhR/Nrf2/NQO1 pathway. Biochem. Pharmacol..

[180] Kempuraj D., Thangavel R., Kempuraj D.D., Ahmed M.E., Selvakumar G.P., Raikwar S.P., Zaheer S.A., Iyer S.S., Govindarajan R., Chandrasekaran P.N. (2021). Neuroprotective effects of flavone luteolin in neuroinflammation and neurotrauma. BioFactors.

[181] He B., Nohara K., Park N., Park Y.S., Guillory B., Zhao Z., Garcia J.M., Koike N., Lee C.C., Takahashi J.S. (2016). The Small Molecule Nobiletin Targets the Molecular Oscillator to Enhance Circadian Rhythms and Protect against Metabolic Syndrome. Cell Metab..

[182] Ferreira P.S., Manthey J.A., Nery M.S., Cesar T.B. (2021). Pharmacokinetics and Biodistribution of Eriocitrin in Rats. J. Agric. Food Chem..

[183] Xu P., Deng B., Zhang B., Luo Q., Song G. (2021). Stretch-Induced Tenomodulin Expression Promotes Tenocyte Migration via F-Actin and Chromatin Remodeling. Int. J. Mol. Sci..

[184] Ramos J.E., Al-Nakkash L., Peterson A., Gump B.S., Janjulia T., Moore M.S., Broderick T.L., Carroll C.C. (2012). The soy isoflavone genistein inhibits the reduction in Achilles tendon collagen content induced by ovariectomy in rats. Scand. J. Med. Sci. Sports.

[185] Carroll C.C., Patel S.H., Simmons J., Gordon B.D., Olson J.F., Chemelewski K., Saw S., Hale T.M., Howden R., Sabbaghi A. (2020). The Impact of Genistein Supplementation on Tendon Functional Properties and Gene Expression in Estrogen-Deficient Rats. J. Med. Food.

[186] Sureda A., Sanches Silva A., Sánchez-Machado D.I., López-Cervantes J., Daglia M., Nabavi S.F., Nabavi S.M. (2017). Hypotensive effects of genistein: From chemistry to medicine. Chem. Biol. Interact..

[187] Wang Z., Wang D., Yang D., Zhen W., Zhang J., Peng S. (2018). The effect of icariin on bone metabolism and its potential clinical application. Osteoporos. Int..

[188] Zhang X., Liu T., Huang Y., Wismeijer D., Liu Y. (2014). Icariin: Does it have an osteoinductive potential for bone tissue engineering?. Phytother. Res. PTR.

[189] Ye C., Zhang W., Wang S., Jiang S., Yu Y., Chen E., Xue D., Chen J., He R. (2016). Icariin Promotes Tendon-Bone Healing during Repair of Rotator Cuff Tears: A Biomechanical and Histological Study. Int. J. Mol. Sci..

[190] Li Y., Yao J., Han C., Yang J., Chaudhry M.T., Wang S., Liu H., Yin Y. (2016). Quercetin, Inflammation and Immunity. Nutrients.

[191] Chen S., Jiang H., Wu X., Fang J. (2016). Therapeutic Effects of Quercetin on Inflammation, Obesity, and Type 2 Diabetes. Mediat. Inflamm..

[192] Wong S.K., Chin K.Y., Ima-Nirwana S. (2020). Quercetin as an Agent for Protecting the Bone: A Review of the Current Evidence. Int. J. Mol. Sci..

[193] Hung L.K., Fu S.C., Lee Y.W., Mok T.Y., Chan K.M. (2013). Local vitamin-C injection reduced tendon adhesion in a chicken model of flexor digitorum profundus tendon injury. J. Bone Jt. Surgery. Am. Vol..

[194] Friedman S.L. (2008). Mechanisms of hepatic fibrogenesis. Gastroenterology.

[195] Liang Y., Xu K., Zhang P., Zhang J., Chen P., He J., Fang Y., Zhou Y., Wang J., Bai J. (2020). Quercetin reduces tendon adhesion in rat through suppression of oxidative stress. BMC Musculoskelet. Disord..

[196] Truong V.L., Jun M., Jeong W.S. (2018). Role of resveratrol in regulation of cellular defense systems against oxidative stress. BioFactors.

[197] Li Y.R., Li S., Lin C.C. (2018). Effect of resveratrol and pterostilbene on aging and longevity. BioFactors.

[198] Nunes S., Danesi F., Del Rio D., Silva P. (2018). Resveratrol and inflammatory bowel disease: The evidence so far. Nutr. Res. Rev..

[199] Poulsen M.M., Fjeldborg K., Ornstrup M.J., Kjær T.N., Nøhr M.K., Pedersen S.B. (2015). Resveratrol and inflammation: Challenges in translating pre-clinical findings to improved patient outcomes. Biochim. Biophys. Acta.

[200] De Ligt M., Timmers S., Schrauwen P. (2015). Resveratrol and obesity: Can resveratrol relieve metabolic disturbances?. Biochim. Biophys. Acta.

[201] Szkudelski T., Szkudelska K. (2015). Resveratrol and diabetes: From animal to human studies. Biochim. Biophys. Acta.

[202] Joe A.K., Liu H., Suzui M., Vural M.E., Xiao D., Weinstein I.B. (2002). Resveratrol induces growth inhibition, S-phase arrest, apoptosis, and changes in biomarker expression in several human cancer cell lines. Clin. Cancer Res..

[203] Howitz K.T., Bitterman K.J., Cohen H.Y., Lamming D.W., Lavu S., Wood J.G., Zipkin R.E., Chung P., Kisielewski A., Zhang L.L. (2003). Small molecule activators of sirtuins extend Saccharomyces cerevisiae lifespan. Nature.

[204] Kaeberlein M., McDonagh T., Heltweg B., Hixon J., Westman E.A., Caldwell S.D., Napper A., Curtis R., DiStefano P.S., Fields S. (2005). Substrate-specific activation of sirtuins by resveratrol. J. Biol. Chem..

[205] Shang X., Lin K., Yu R., Zhu P., Zhang Y., Wang L., Xu J., Chen K. (2019). Resveratrol Protects the Myocardium in Sepsis by Activating the Phosphatidylinositol 3-Kinases (PI3K)/AKT/Mammalian Target of Rapamycin (mTOR) Pathway and Inhibiting the Nuclear Factor-κB (NF-κB) Signaling Pathway. Med. Sci. Monit. Int. Med. J. Exp. Clin. Res..

[206] Constanze B., Popper B., Aggarwal B.B., Shakibaei M. (2020). Evidence that TNF-β suppresses osteoblast differentiation of mesenchymal stem cells and resveratrol reverses it through modulation of NF-κB, Sirt1 and Runx2. Cell Tissue Res..

[207] Poulsen R.C., Watts A.C., Murphy R.J., Snelling S.J., Carr A.J., Hulley P.A. (2014). Glucocorticoids induce senescence in primary human tenocytes by inhibition of sirtuin 1 and activation of the p53/p21 pathway: In vivo and in vitro evidence. Ann. Rheum. Dis..

[208] Mikolyzk D.K., Wei A.S., Tonino P., Marra G., Williams D.A., Himes R.D., Wezeman F.H., Callaci J.J. (2009). Effect of corticosteroids on the biomechanical strength of rat rotator cuff tendon. J. Bone Jt. Surgery. Am. Vol..

[209] Selvakumar G., Venu D., Kuttalam I., Lonchin S. (2022). Inhibition of Advanced Glycation End Product Formation in Rat Tail Tendons by Polydatin and p-Coumaric acid: An In Vitro Study. Appl. Biochem. Biotechnol..

[210] Riva A., Allegrini P., Franceschi F., Togni S., Giacomelli L., Eggenhoffner R. (2017). A novel boswellic acids delivery form (Casperome^®^) in the management of musculoskeletal disorders: A review. Eur. Rev. Med. Pharmacol. Sci..

[211] Franceschi F., Togni S., Belcaro G., Dugall M., Luzzi R., Ledda A., Pellegrini L., Eggenhoffner R., Giacomelli L. (2016). A novel lecithin based delivery form of Boswellic acids (Casperome^®^) for the management of osteo-muscular pain: A registry study in young rugby players. Eur. Rev. Med. Pharmacol. Sci..

[212] Merolla G., Dellabiancia F., Ingardia A., Paladini P., Porcellini G. (2015). Co-analgesic therapy for arthroscopic supraspinatus tendon repair pain using a dietary supplement containing Boswellia serrata and Curcuma longa: A prospective randomized placebo-controlled study. Musculoskelet. Surg..

[213] Vitali M., Naim Rodriguez N., Pironti P., Drossinos A., Di Carlo G., Chawla A., Gianfranco F. (2019). ESWT and nutraceutical supplementation (Tendisulfur Forte) vs. ESWT-only in the treatment of lateral epicondylitis, Achilles tendinopathy, and rotator cuff tendinopathy: A comparative study. J. Drug Assess..

[214] Notarnicola A., Pesce V., Vicenti G., Tafuri S., Forcignanò M., Moretti B. (2012). SWAAT study: Extracorporeal shock wave therapy and arginine supplementation and other nutraceuticals for insertional Achilles tendinopathy. Adv. Ther..

[215] Gumina S., Passaretti D., Gurzì M.D., Candela V. (2012). Arginine L-alpha-ketoglutarate, methylsulfonylmethane, hydrolyzed type I collagen and bromelain in rotator cuff tear repair: A prospective randomized study. Curr. Med. Res. Opin..

[216] Notarnicola A., Maccagnano G., Tafuri S., Fiore A., Pesce V., Moretti B. (2015). Comparison of shock wave therapy and nutraceutical composed of Echinacea angustifolia, alpha lipoic acid, conjugated linoleic acid and quercetin (perinerv) in patients with carpal tunnel syndrome. Int. J. Immunopathol. Pharmacol..

[217] Poeckel D., Werz O. (2006). Boswellic acids: Biological actions and molecular targets. Curr. Med. Chem..

[218] Siddiqui M.Z. (2011). Boswellia serrata, a potential antiinflammatory agent: An overview. Indian J. Pharm. Sci..

[219] Sharifi-Rad M., Mnayer D., Morais-Braga M.F.B., Carneiro J.N.P., Bezerra C.F., Coutinho H.D.M., Salehi B., Martorell M., Del Mar Contreras M., Soltani-Nejad A. (2018). Echinacea plants as antioxidant and antibacterial agents: From traditional medicine to biotechnological applications. Phytother. Res. PTR.

[220] Shi Q., Lang W., Wang S., Li G., Bai X., Yan X., Zhang H. (2021). Echinacea polysaccharide attenuates lipopolysaccharide-induced acute kidney injury via inhibiting inflammation, oxidative stress and the MAPK signaling pathway. Int. J. Mol. Med..

[221] Zhang H., Lang W., Wang S., Li B., Li G., Shi Q. (2020). Echinacea polysaccharide alleviates LPS-induced lung injury via inhibiting inflammation, apoptosis and activation of the TLR4/NF-κB signal pathway. Int. Immunopharmacol..

[222] Steinmann S., Pfeifer C.G., Brochhausen C., Docheva D. (2020). Spectrum of Tendon Pathologies: Triggers, Trails and End-State. Int. J. Mol. Sci..

[223] Sharip A., Kunz J. (2020). Understanding the Pathogenesis of Spondyloarthritis. Biomolecules.

[224] Mueller A.L., Payandeh Z., Mohammadkhani N., Mubarak S.M.H., Zakeri A., Alagheband Bahrami A., Brockmueller A., Shakibaei M. (2021). Recent Advances in Understanding the Pathogenesis of Rheumatoid Arthritis: New Treatment Strategies. Cells.

[225] Abramoff B., Caldera F.E. (2020). Osteoarthritis: Pathology, Diagnosis, and Treatment Options. Med. Clin. N. Am..

[226] Sari Z., Aydoğdu O., Demirbüken İ., Yurdalan S.U., Polat M.G. (2019). A Better Way to Decrease Knee Swelling in Patients with Knee Osteoarthritis: A Single-Blind Randomised Controlled Trial. Pain Res. Manag..

